# Automated reaction database and reaction network analysis: extraction of reaction templates using cheminformatics

**DOI:** 10.1186/s13321-018-0269-8

**Published:** 2018-03-09

**Authors:** Pieter P. Plehiers, Guy B. Marin, Christian V. Stevens, Kevin M. Van Geem

**Affiliations:** 1 Laboratory for Chemical Technology, Department of Materials, Textiles and Chemical Engineering, Ghent University, Technologiepark 914, 9052 Ghent, Belgium; 2 SynBioC Research Group, Department of Sustainable Organic Chemistry and Technology, Faculty of Bioscience Engineering, Ghent University, Coupure Links 653, 9000 Ghent, Belgium

**Keywords:** Cheminformatics, Reaction network, Synthesis planning, Reaction template

## Abstract

Both the automated generation of reaction networks and the automated prediction of synthetic trees require, in one way or another, the definition of possible transformations a molecule can undergo. One way of doing this is by using reaction templates. In view of the expanding amount of known reactions, it has become more and more difficult to envision all possible transformations that could occur in a studied system. Nonetheless, most reaction network generation tools rely on user-defined reaction templates. Not only does this limit the amount of chemistry that can be accounted for in the reaction networks, it also confines the wide-spread use of the tools by a broad public. In retrosynthetic analysis, the quality of the analysis depends on what percentage of the known chemistry is accounted for. Using databases to identify templates is therefore crucial in this respect. For this purpose, an algorithm has been developed to extract reaction templates from various types of chemical databases. Some databases such as the Kyoto Encyclopedia for Genes and Genomes and RMG do not report an atom–atom mapping (AAM) for the reactions. This makes the extraction of a template non-straightforward. If no mapping is available, it is calculated by the Reaction Decoder Tool (RDT). With a correct AAM—either calculated by RDT or specified—the algorithm consistently extracts a correct template for a wide variety of reactions, both elementary and non-elementary. The developed algorithm is a first step towards data-driven generation of synthetic trees or reaction networks, and a greater accessibility for non-expert users.

**Electronic supplementary material:**

The online version of this article (10.1186/s13321-018-0269-8) contains supplementary material, which is available to authorized users.

## Background

The continuous increase of our scientific knowledge has led to data quantities that can no longer be processed by the human mind alone: the Reaxys^®^ database contains over 40 million chemical reaction entries and lists over 100 million compounds [1]. With computers being increasingly used for discovering new chemistry and improving our knowledge of existing chemistry, the rate of this expansion will only increase in the future. Computer-aided discovery has been adopted in drug discovery [2–4] and has found uses in other fields, such as geography and astronomy [5]. The recent revival of interest in retro-synthetic analysis [6, 7] is also an example of computational enhancement of our scientific knowledge.

**Fig. 1:**
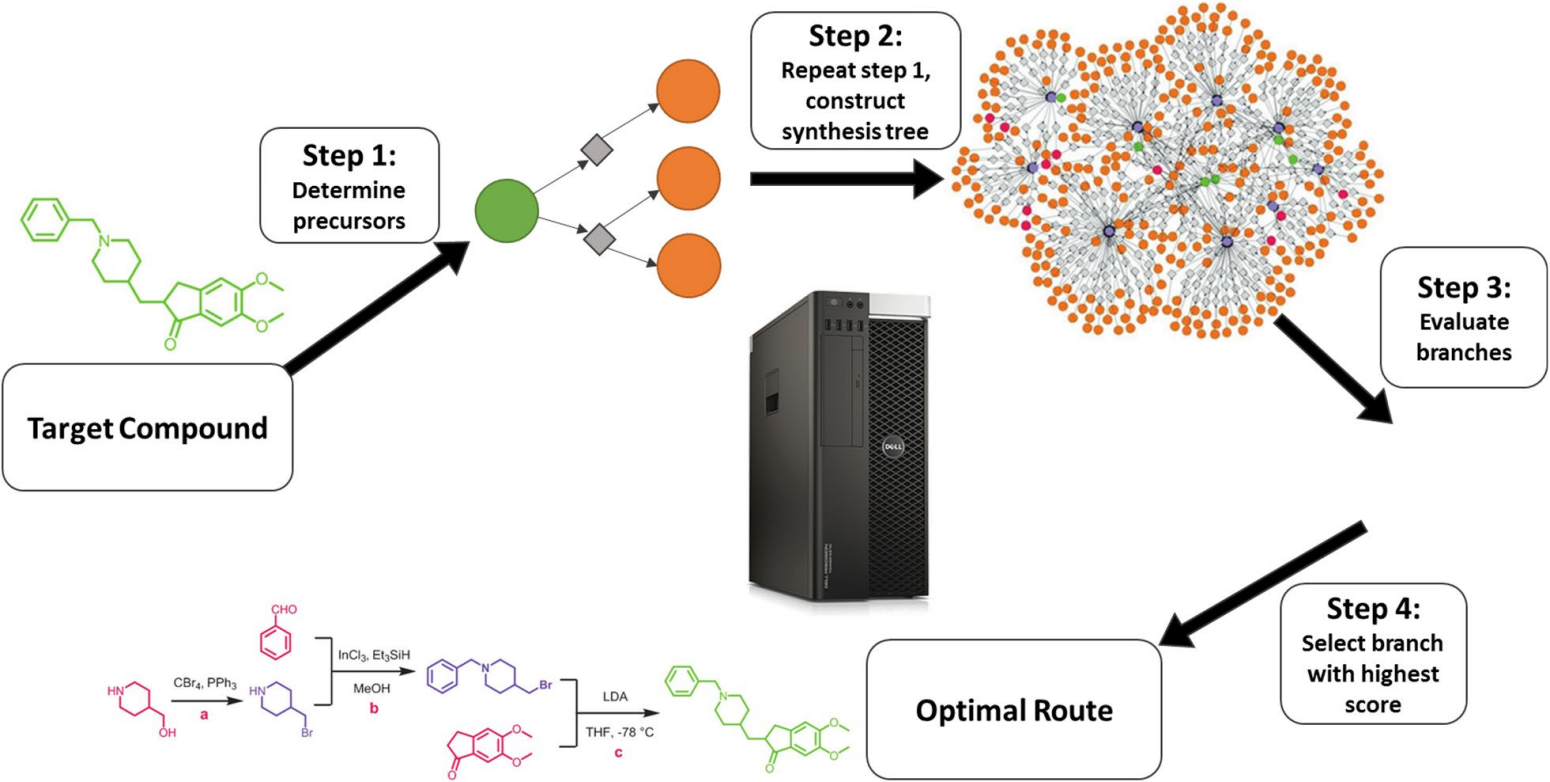
Simplified, general flow scheme of retro-synthetic software. In the first step, a series of possible precursor molecules is determined for the target molecule. In the second step, iterative application of the first step to each new precursor results in the construction of a synthesis tree. The next step assigns a score to each path in the obtained synthesis tree according to some scoring function. Ranking of all possible routes based on this score finally results in the selection of optimal synthetic pathway for the specified target compound

**Fig. 2:**
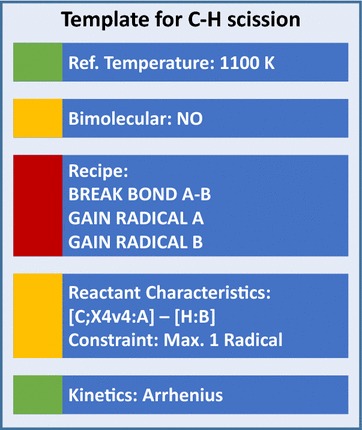
Illustration of a reaction template based on the example of the C–H bond scission, indicating the different types of information contained in it: molecular characteristics of the reactants, required for the reaction to take place (yellow); the recipe—changes that take place during the reaction (red), additional information such as kinetics and reference temperature (green)

**Fig. 3:**
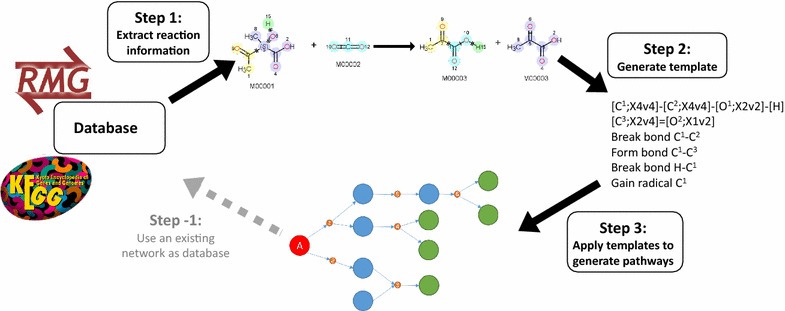
From reaction database entry to reaction network: the first step consists of extracting information for each reaction in the database, such as the atom–atom mapping. The second step analyzes this information and converts it into a reaction template. A number of these templates can be used in a third step to generate a synthesis tree or a reaction network. A CHEMKIN^®^ network is a possible data source as well. This results in the possibility of using such a network to construct a database as preprocessing step

Chemical databases indisputably contain a tremendous amount of potentially useful information for both automated retro-synthetic analysis tools and kinetic model generation tools. The overall concept of a retro-synthetic tool is illustrated in Fig. 1, showing that one of the steps is generating a synthesis tree [6]. This requires knowledge of which transformations a given molecule can undergo. One way of defining these transformations is by using reaction templates: generalized blueprints of a reaction that determine which type of substructures are required in the reactants and provide a recipe for how the reactants are transformed into the products. Similar information is required in kinetic model generation tools—another example of computer-aided knowledge enhancement. Tools such as Genesys [8, 9] and RMG [10], successfully make use of reaction templates that are iteratively applied to all species in the network. Besides defining required molecular characteristics and the recipe, they also potentially provide important information on the reaction kinetics. A schematized example of a template as used in Genesys is shown in Fig. 2. In most network generators, the templates are constructed manually, which can be a tedious process. In the current trend towards using extensive databases or existing, detailed chemical reaction networks as a source from which such recipes can be extracted, this step could be eliminated allowing for more efficient and complete reaction network generation as shown in Fig. 3. Manual enumeration of possible templates might be feasible for the generation of a reaction network for a system in which a limited number of reaction types takes place, such as pyrolysis. The accuracy of a retro-synthetic tool on the other hand, relies heavily on the extent of the chemical knowledge that is incorporated within, making it impossible to achieve high accuracy while manually enumerating all probable reaction templates. The ability to extract templates automatically from extensive databases is therefore of great importance in the development of a retro-synthesis tool.

In this work, several database sources are considered. To represent organic, liquid phase chemistry and potential application in retro-synthesis, the Kyoto Encyclopedia for Genes and Genomes (KEGG) [11] is used. The RMG kinetics database [12] and CHEMKIN^®^ format reaction networks [13] are used as representatives for pyrolysis/combustion chemistry and potential use in reaction network generation. The methods described hereafter have been designed for the aforementioned reaction types. Therefore, the method will not perform optimally for solid phase chemistry, polymerization chemistry and systems with interface chemistry such as heterogeneously catalyzed reactions.

**Fig. 4:**
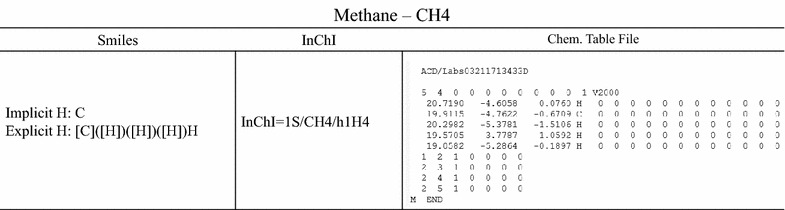
Three standardized chemical identification formats from left to right: Smiles, InChIs and chemical table

Using databases presents several challenges. A first problem that is encountered is that reaction databases, are often incomplete and/or use non-standardized nomenclature for their species. Examples of the latter are the use of trivial names, or chemical formulas from which it is very difficult to derive the correct structure as they contain little or no standardized information on the connectivity of the atoms. Fortunately, most databases do link a standardized identifier to each species. The most frequently used identifiers are the International Chemical Identifier (InChI) [14] and the Simplified Molecular Input Line System (SMILES) [15] which are illustrated in Fig. 4. Both formats were developed to be machine readable. The SMILES format is also easily human readable, but is not unique. Via canonicalization of the molecule [16] it is possible to derive a standardized version of the SMILES. This problem is not encountered with the InChI format. The InChI algorithm ensures that one molecule is identified by one InChI and vice versa, but makes it very difficult to be interpreted by a human. This uniqueness allows fast comparison of molecules, without having to resort to time-consuming maximum common subgraph isomorphism tests [17]. Another identification format that is relevant in this work, are chemical table files [18]. The format represents a molecule by listing the connections between the different atoms and is illustrated in Fig. 4. A chemical table file consists of two main parts. A first part lists all the atoms and their characteristics such as charge and multiplicity. The second part describes all bonds in the molecule. Some chemical table file-derived formats allow an additional property block. Finally, it is worth mentioning the Smiles Molecular Arbitrary Target Specification (SMARTS) [19], which is an extension of the SMILES format to allow identification of molecular fragments.

A second problem with databases is the lack of a complete description of the reactions. Reactants or products can be missing, resulting in unbalanced reactions and there is often no information on the atom–atom mapping (AAM) of the reactant atoms to the product atoms. The AAM links reactant and product atoms, i.e. identifies which product atoms originate from which reactant atoms. A wide variety of algorithms exist to determine the AAM of reactions [20]. The two most employed algorithms are those based on finding the maximum common substructure (MCS) between the reactants and products [21, 22] and those optimizing some constrained cost function. Examples of the latter are the mixed-integer linear optimization approach [23, 24] and minimizing the edit distance [25] or the energy of the imaginary transition state [26, 27]. Without going into further detail on these algorithms, it should be noted that finding an AAM solution is a computationally expensive process that is not without error [20].

A final comment is reserved for the kinetics of database entries. In some cases, such as KEGG, no kinetic data is available. In others, such as RMG, kinetic data is given for specific reactions. With the databases sometimes containing only a limited number of reactions representing the same reaction family, it is difficult to generalize this data to be applicable on other members of that reaction family. The same is true for a CHEMKIN^®^ network, though here the amount of reactions belonging to the same reaction family is typically higher. As reaction network generation tools often use group additivity [28] to determine kinetics for the different reactions [8, 9], a future effort could be to derive these values from the kinetic parameters in the reaction network.

In what follows, we will describe a tool and relevant algorithms that allow the user to extract reaction templates from databases of various formats. The current focus is on extracting templates that can be used for the generation of reaction networks or the prediction of retro-synthetic trees, i.e. to determine which products are formed from certain reactants. To arrive to a kinetic model will still require manual specification of kinetics. While the idea of automated reaction template extraction is not new, it has mainly been mentioned on the side in other topics [29–31]. It is—to our knowledge—the first time a stand-alone application is published. Moreover, previous methods focused mainly on the extraction from specific databases, whereas our methods attempts at being more flexible in its input handling. The possibility to determine AAMs where necessary contributes to this flexibility.

In the "Algorithm" section, the specificities of the algorithm and sub-algorithms are discussed. The "Results and discussion" section describes the results of applying the extraction method to three different cases. A first case represents a database of radical chemistry, the second one of liquid phase chemistry and the final is the analysis of a full reaction network. Some final conclusions and thoughts are presented in the "Conclusions" section.

## Algorithm

**Fig. 5:**
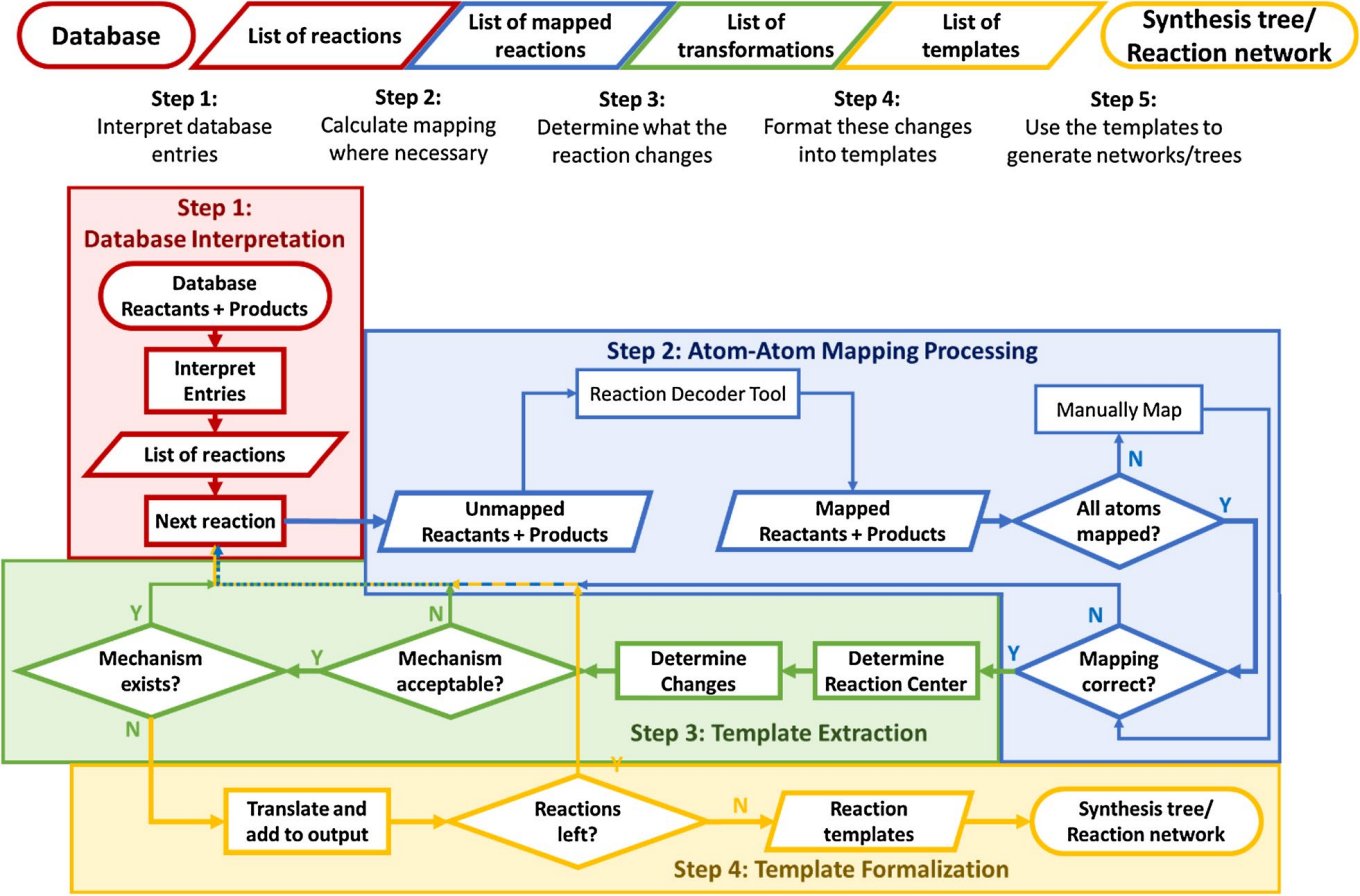
Schematic overview of the reaction template extraction algorithm. The general scheme is shown at the top of the figure, the scheme below details each step further. Each color groups blocks belonging to one sub-task: database interpretation (red), mapping (blue), extraction (green) and post-processing (yellow)

**Fig. 6:**
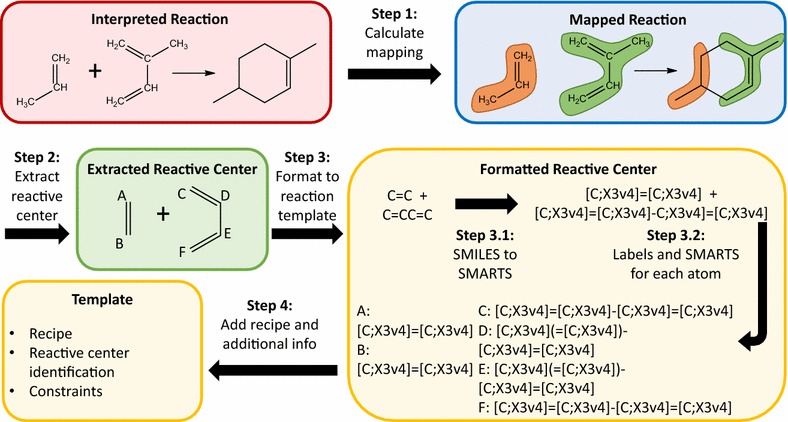
Illustration of the identification of a reactive center for the Diels–Alder reaction. The colors refer to the general section of the algorithm where the step is performed: database interpretation (red), mapping (blue), extraction (green) and formalizing the template (yellow). Starting from the interpreted reaction, the first two steps allow for the extraction of the reactive center. Step 3 formalizes the identification of the reactive center for the template. First the SMILES identifier is generated. In a second step the number of neighbors (‘X3’ implies three neighbors), the valence (v4 implies a valence of 4, i.e. a non-radical carbon atom) of each atom are added to the identifier to arrive at the SMARTS identifier. Finally SMARTS and labels are generated for each of the atoms in the reactive center, with the described atom listed first. This information is then combined with the recipe for the reaction into the template

The algorithm can be divided into four major processes, as schematically illustrated in Fig. 5. Each of the steps will be discussed in more detail in what follows. Figure 6 illustrates the algorithm using the example of the Diels–Alder reaction between propene and 2-methyl-butadiene. Each frame of the figure shows the outcome of each of the steps in Fig. 5.

The open source cheminformatics package “The Chemical Development Kit” (CDK) [32] is used as platform for the representation and manipulation of chemical entities. The employed version is an independently developed branch of CDK v1.4.11, which has been fine-tuned to the requirements of Genesys. Other open source chemical software packages that are incorporated are JNI-InChI v0.7 [33] for the generation of InChI identifiers and AMBIT-SMARTS [19] for SMARTS processing.

### Database interpretation

The interpretation and pre-processing of database information is the first step in the algorithm and corresponds to the part of the scheme in Fig. 5 that is outlined in red.

To allow for flexibility in handling different databases, several input formats have been implemented. There are three different file types from which reactions can be read. The first is a structured text file listing SMILES or InChIs for the reactants and products of each reaction. Input is also possible in the form a directory containing Molecular Design Ltd. (MDL) reaction (.rxn) files [18], which are based on the chemical table files. This format is the preferred type as it allows for easy storage of AAMs. All reactions in the two databases considered (KEGG and RMG) are first preprocessed into the MDL reaction format in order to have a single input type. In KEGG this is done simply by joining the separate chemical table files of each molecule that participates in the reaction. In RMG the listed InChIs of the reactants and products are used to construct the files. Should for any reason no InChI be present for a molecule, the listed SMILES representation is used. A final input format are CHEMKIN^®^ input files. CHEMKIN^®^ does not require species to be identified by any systematic name. This makes interpreting these species very difficult [34]. Therefore, the current implementation requires the user to provide an identifier of choice (InChI or SMILES) as comment to each species. Based on these identifiers, molecules are assigned to the user-defined names and the reactions in the network are interpreted. As mentioned above, the reaction rate coefficients are not further considered as the focus is on extracting the reaction template from the reaction. It is up to the user to specify the desired kinetics if a kinetics model will be generated form the templates.

To ensure compatibility with Genesys while testing, unbalanced reactions are filtered out.

### Atom–atom mapping

The next step consists of determining an AAM for each of the retained reactions from the database and corresponds to the blue section in Fig. 5.

**Fig. 7:**
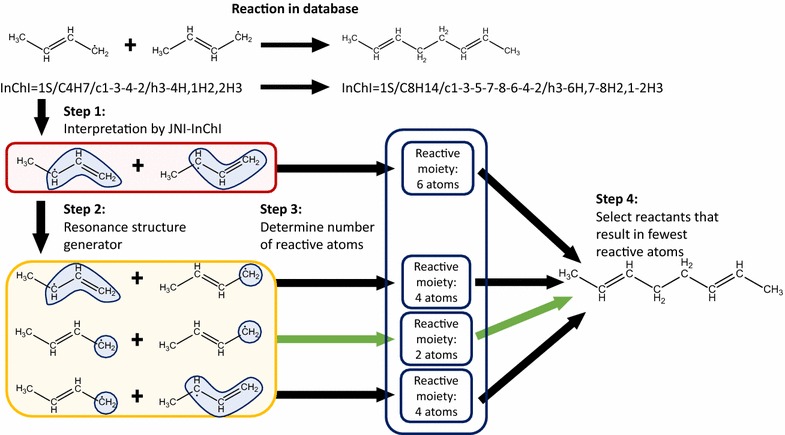
Illustration of how the correct resonance structure is found for template extraction. The example shows the addition of two, resonance stabilized butenyl radicals. In order to form the shown product, the reaction should take place between two primary radicals. In the first step, the database identifier is interpreted. The algorithm of JNI-InChI returns the secondary radical. The next step generates all possible combinations of electron localizations. In the third step, the number of atoms that make up the reactive moiety is determined for each combination. Finally, the combination that gives rise to the fewest reactive atoms is chosen as correct, localized representation of the reaction in the database

Several tools have been developed to calculate the AAM of a reaction, though few of them have open access. Examples of protected software are REACCS by Accelrys [35] and DREAM by Princeton [23]. Adding a preference for the use of the CDK as supporting cheminformatics software to optimize compatibility, two open-source tools have been found. The first has been developed by Crabtree et al. and provides a graphical user interface (GUI) [36]. The second is the Reaction Decoder Tool (RDT), developed by Rahman et al. [37, 38]. The latter reports excellent accuracies, uses the preferred MDL.rxn files for reporting and provides source code access. Therefore, the RDT is chosen for performing the AAM in this tool. The RDT is based on a MCS algorithm, but applies four different variants to find the most optimal AAM, i.e. the one with minimal structural- and bond changes. Some minor adaptations to the RDT have been made to allow for the processing of reactions containing radical species. The main adaption is implemented in the chemical format parser of the RDT. A correction on the determination of the valence and neighbor counts for each atom was required to correctly take radicals into account. Input to the RDT requires a localized definition of resonance structures. For molecules in which resonance is detected, the mapping is performed for each possible combination of resonance structures, as both the detected mapping and the extracted template can be different depending on the considered localized resonance structure. The possible resonance structures are automatically generated via the CDK. This is illustrated by Fig. 7. Only the combination of resonance structures that results in the lowest number of atoms whose connectivity has changed, is retained. This is of particular importance when molecules have been input via InChI identifiers, which do not distinguish between resonance structures. From an InChI, JNI-InChI generates a molecule in which all electrons are localized. Depending on the number of possible resonance structures, this results in a limited chance that the structure that results in a minimal number of changes by the reaction, is obtained.

**Fig. 8:**
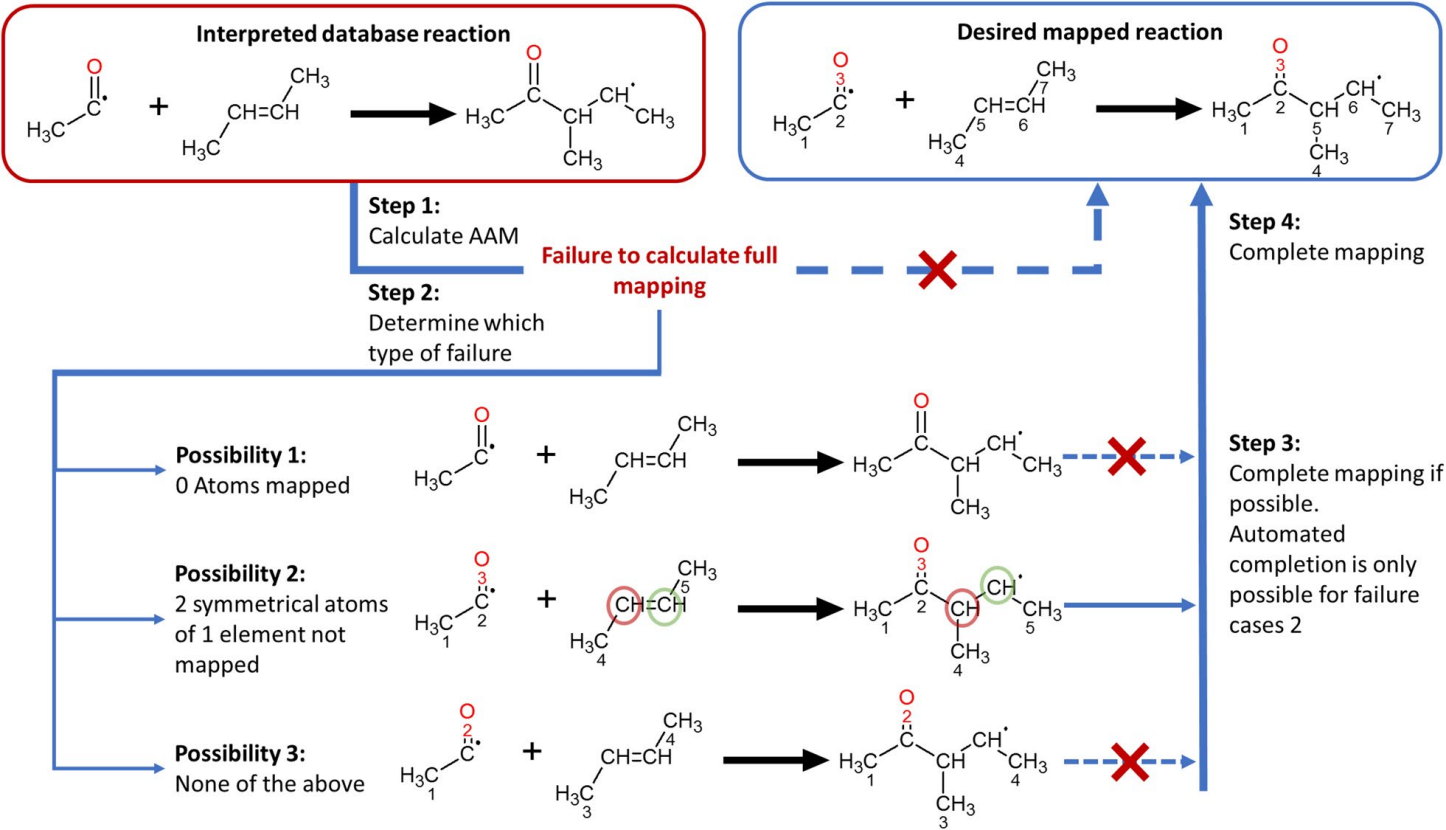
Heuristic approach to completing calculated mappings. The colored circles indicate which atoms can be heuristically mapped to each other. If the mapping calculation fails to determine a full mapping, the failures can be categorized in 3 classes. The first possibility is that no atoms have been mapped; no heuristic completion is possible here. In the second, two atoms of the same element are not mapped. In case of symmetry of the reactant or product, only one possible mapping remains: red on red and green on green. All other failures are categorized into the third class, for which no completion is possible

**Fig. 9:**
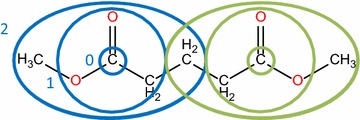
Subgraphs of radius n. The subgraph containing only the root atom has radius 0. Each subgraph of radius n + 1 contains all atoms connected to all atoms of the subgraph with radius n. In this example the carbon atoms around which the subgraphs are centered would be considered to be chemically identical

**Fig. 10:**
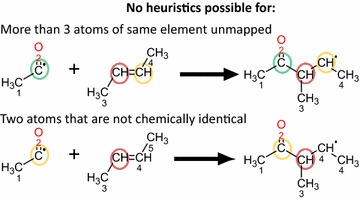
Forbidden combinations of unmapped atoms for heuristic completion. Circles of same color indicate which atoms should be mapped to each other. The first example combines two atoms that are in identical chemical positions and one lone unmapped atom. This combination cannot be corrected with certainty of not altering the template. In the second case, two atoms are unmapped, but they do not have chemically identical environments in either reactants or products

Once the mapping has been determined, a check is run on the mapping. As the RDT has been designed with a focus on organic reactions and all algorithms of the RDT rely on substructure matching, incomplete or incorrect mappings can be expected for small molecules. Especially for reactions such as combustion and pyrolysis of C_1_–C_2_ hydrocarbons, problems can be encountered. There is a specific case in which the mapping is incomplete, but for which a method has been devised to complete it. The case handling for this amendment is illustrated by Fig. 8. For some reactions, two atoms of the same element have not been mapped and both atoms are present in identical chemical surroundings, i.e. there is some form of symmetry present. Note that these identical surroundings can be found in either the reactants or products. To assess the similarity of environments of a molecule we rely on a molecular graph equivalent of the eccentricity of a vertex in graph theory [39]. The radius of a molecular subgraph centered on a certain root atom, is the minimal number of bonds between the root atom and the terminal atoms, as is illustrated in Fig. 9. The radius of a molecule, relative to a given root atom is found by extending the subgraph until it contains the entire molecule. Two atoms are considered to be in identical chemical environments if one of the following is true. If they belong to the same molecule, they are considered identical if the molecule has the same radius (r) relative to each of the atoms and all subgraphs of radius r − i (i = 1..r) are isomorphic. This corresponds to the molecule having an axis of symmetry between the considered atoms. If the atoms belong to two different molecules, they are considered identical if all subgraphs of radius r − i (i = 0..r) around both atoms are isomorphic. This corresponds to the atoms being in the same chemical position in two identical molecules. In the case described above, the two remaining atoms are mapped such that they retain as many neighbors as possible from the reactant molecule. In other cases such as those presented in Fig. 10, it is not possible to heuristically complete the mapping.

A final step in the AAM section is a check on the mapping correctness. Assessment of the correctness of the mapping is done based on the completeness of the mapping.

### Recipe extraction

Based on the mapping of reactant atoms to product atoms, the reactive center can now be extracted. In Fig. 5, this section is outlined in green.

**Fig. 11:**
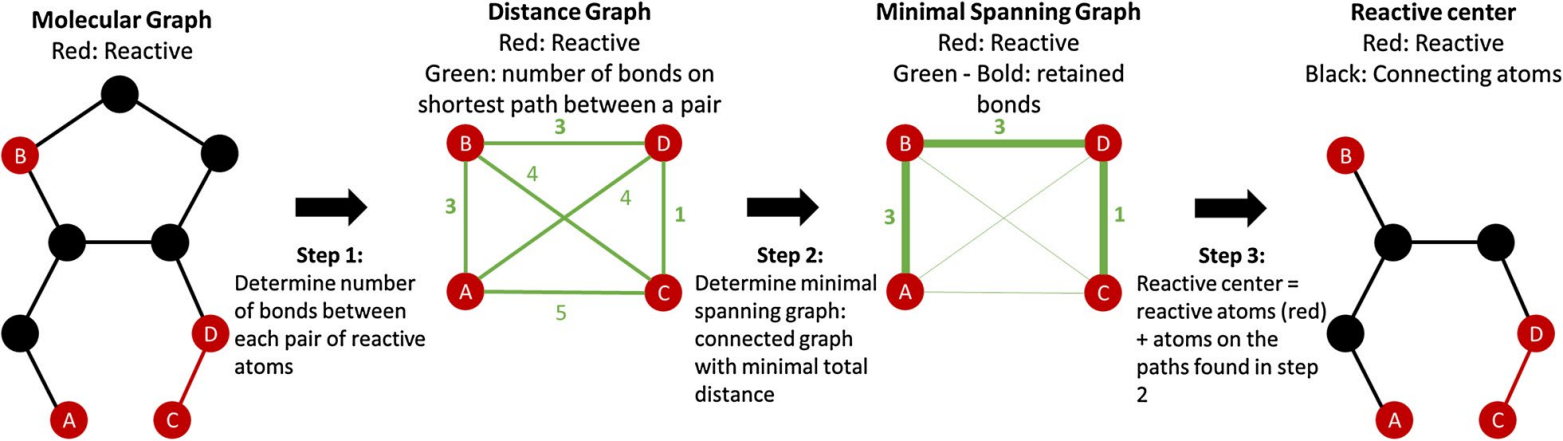
Generic example of the algorithm to construct a connected graph for disconnected reaction centers. Red atoms are reactive atoms whose environment is changed by the reaction, black ones have no change in environment. The first step finds the shortest path between each pair of two reactive atoms. The number of bonds in these paths are used to construct the connected distance graph. In a second step, the minimal spanning graph is determined from which the smallest connected graph of the reactive atoms is constructed in the final step

First, the reactive center is extracted. This is done by identifying which atoms’ environments have been changed by the reaction. Table 1 describes in which cases the environment of an atom is considered to have been changed by the reaction and how the change is detected. This leads to the formation of the so-called reactive center, which only contains the atoms with changed environments. In case of intramolecular reactions, it is possible that the remaining atoms do not form a connected graph. The way the reactive atoms are connected can be of importance not to overgeneralize the template though. Therefore, atoms must be added to the reactive center to ensure a connected graph is obtained. The consistent identification of these connecting atoms is important as they will influence how successfully reaction templates are compared. The different steps are illustrated in Fig. 11. First the shortest path is determined between each of the identified atoms via a breadth first search. This results in a new connected and weighted graph, with the atoms as nodes and the number of bonds in the shortest path between them as weighted edges. A minimal spanning tree of this graph [40] is determined to find which of the connections between the atoms result in the minimal number of bonds in the final extended reactive center. All atoms that are present in the selected paths are also added to the reactive center, but with the label that they are only added to ensure the connectivity of the reactive center. A user option is available to further expand all reactive centers by including hetero-atoms connected to the reacting atoms. This makes the final reactive center more specific and can be useful if surrounding atoms influence or are required in the electronic transitions that occur during the reaction, without changing on a net basis.

**Table 1:** Overview of possible changes to the environment of an atom. Changes are detected by comparing atoms that are mapped to each other. In each example case, the changes in the environment of the blue atom are assessed. This is done by either comparing the two blue atoms directly (for radicals, charges and stereo), or by comparing the surroundings (bond changes)

Changes in environment	Example	Description
Losing/gaining neighbors		Comparing neighbors of ‘A’ and their bonds to ‘A’ to those of ‘A’s mapped counterpart. A match is found if all neighboring element names are equal and all neighboring mapping indices are equal
Changing bond orders		Comparing the order of ‘A’s bonds to those of ‘A’s mapped counterpart. Marked if the bond order between ‘A’ and a neighbor has changed
Losing/gaining radicals		Comparing single electron count of ‘A’ to its mapped counterpart
Losing/gaining charge		Comparing charge on ‘A’ to that on ‘A’s mapped counterpart
Changing stereo configuration		Comparing E/Z or R/S configuration of ‘A’ to that of its mapped counterparts

**Fig. 12:**
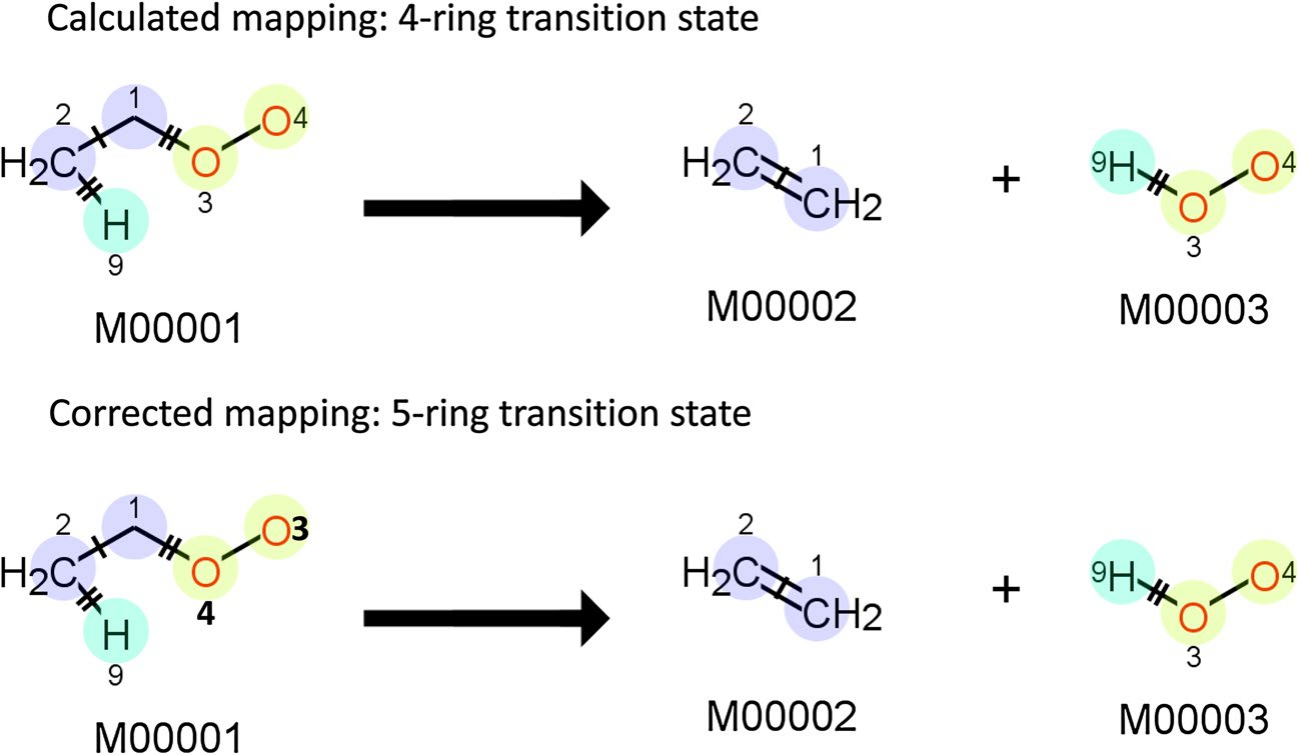
Top—mapping found by the RDT for the elimination of HOO^•^. The numbers indicate which reactant atom has been mapped to which product atom. Bottom—corrected mapping to represent a five-ring transition state

Once the changes to the reactant atoms have been determined, a heuristic check of mechanism acceptability is performed. It has been observed that for some radical reactions, the mapping is not determined correctly. In all these cases, the mapping was such that no change occurred to the radical; it did not participate in the reaction mechanism. As reactions involving radicals typically react via the radical, it is assumed that these mappings are incorrect. Such mappings are not further processed, with one exception. The elimination of a hydrogen peroxide (HOO^•^) from a peroxide (ROO^•^) to form an olefin is consistently misinterpreted by the RDT, as shown in Fig. 12. The mapping determined by the RDT suggests a four-ring cyclic transition state. Based on work by Li [41], it is much more likely that the reaction will occur via a five-ring transition state, so the mapping for this type of reactions is adjusted. This also highlights one of the drawbacks of using computed AAMs; even a seemingly correct mapping does not always result in the correct template. One could argue that using the imaginary transition state energy approach of Körner et al. [26] could resolve this error. Their method will indeed detect both the four- and five-membered ring mappings, but it will not be able to indicate which one is actually preferred as their calculation of the transition state energy does not account for ring strain. The increase in complexity incurred by introducing such contributions does not weigh up to their benefit in the few cases in which they will influence the mapping, not in the least because these cases are easily recognized and corrected.

To limit the amount of stored data, only unique reaction templates are retained for further processing. Two reaction templates are considered equal if the reactive centers are equal and all of the detected changes are equal. Besides checking templates for uniqueness, they are also checked for reversibility. Templates are considered the reverse of each other if the products of one are equal to the reactants of the other and vice versa. Additionally, application of the detected changes to the reactants of one template should result in the reactants of its reverse counterpart. This definition will not identify reverse reactions correctly with 100% certainty. Consider the 1–2 hydrogen shift in 1-pentyl. This results in the 2-pentyl radical. Later in the database, the 1–4 hydrogen shift in 2-pentyl is encountered. This results in the 1-pentyl radical. Based on the above definition of reverse reaction templates and a correct AAM, this would result in them being considered the reverse of each other. The concept of reverse reactions is of specific importance if kinetics are intended to be calculated. As mentioned in the “Background” section though, the manual assignment of the kinetics will require a great deal of time and effort, at which point the incorrect labels are easily corrected. A second problem is illustrated by the following example. If the AAM for the previously mentioned reactions has to be calculated, the RDT will calculate the wrong mapping for the 1–4 shift, the found mapping will result in a template describing the 1–2 shift. Similarly the 1–4 shift in 2-hexyl will incorrectly be interpreted as an identical reaction. From these two examples it is clear that symmetry of reactants and products can introduce mapping errors that are very difficult to notice. It should be noted that in the general context of extracting templates from a large database, this mapping error will typically not result in extracting wrong templates or missing out on any. If the 1–4 hydrogen shift in 2-pentyl is present in the database, it is not unreasonable to expect e.g. the 1–4 shift in 1-heptyl to be present as well. For the latter case, the correct template for a 1–4 hydrogen shift is extracted. Only in the analysis of how many times a template is encountered will there remain an error. As the primary goal of the tool is to extract templates, this counting issue is not considered critical.

### Formalizing the template

The final step prior to generating a network based on the data, is post-processing the accumulated data into Genesys-readable content. This last step is colored yellow in Fig. 5.

For each reaction template that has been determined, four input elements are generated; the recipe, the definition of the reactants, molecular constraints for the rule-based algorithm of Genesys and kinetics. The recipe is simply assigning the correct labels to each change that belongs to the reaction template. Defining the reactants requires a SMARTS identifier for the reactive center and for each atom that participates in the reaction. A custom SMARTS generator has been developed to fulfill all Genesys-specific requirements for the reactive center identification. As mentioned in the introduction, SMARTS is an extension to the SMILES format. This implies that any SMILES identifier is a valid SMARTS identifier, which makes the SMILES of the extracted reactive center a good starting point for its SMARTS. The reactive center is specified in greater detail by adding valences and neighbor counts to each atom, re-identifying aromatic atoms and making all bonds explicit. This procedure is illustrated in the yellow section of Fig. 6. A second part of the reaction center identification is identifying the individual reacting atoms. This requires an additional step in the generation of the SMARTS identifier as identification of an atom in a certain environment via SMARTS requires the identified atom to be written first. The order in which the atoms in the (non-canonical) SMILES are written, depends on the order in which the atoms are stored internally. The to-be defined atom is moved to the first position by permuting the order of the atoms until the identifier starts with the desired atom, after which the SMARTS can be generated. Related to defining the reactant templates is defining appropriate constraints for the network generator. Two different constraints are generated automatically. The first is a global constraint that limits the molecule size to that of the largest molecule encountered in the analyzed database. The second is reactant dependent and limits the number of single electrons to the number of single electrons in the reactant. Other constraints can be added by the user afterwards. Finally, the kinetics element is generated. As mentioned previously, the focus of the method is on extracting the reaction template, in order to generate a network, not a kinetic model. Therefore, an empty block for group additivity based kinetics calculations is added, with the user having to fill out the path to the desired database of ΔGAV^0^s [42–44].

## Results and discussion

### KEGG

A subset from the KEGG database is analyzed. The entries R00002–R01500 are selected, totaling 1110 reactions. 263 of them involve three or more reactants. The network generation tool Genesys has been programmed to process reactions with one or two reactants. As the derived templates will be tested using Genesys, the reactions with more than two reactants are excluded from the analysis, leaving 847 reactions. For the sake of the analysis, only the forward reactions are considered. The exact reaction ids can be found in the supporting information (Additional file 3) in section 1.1.

**Fig. 13:**
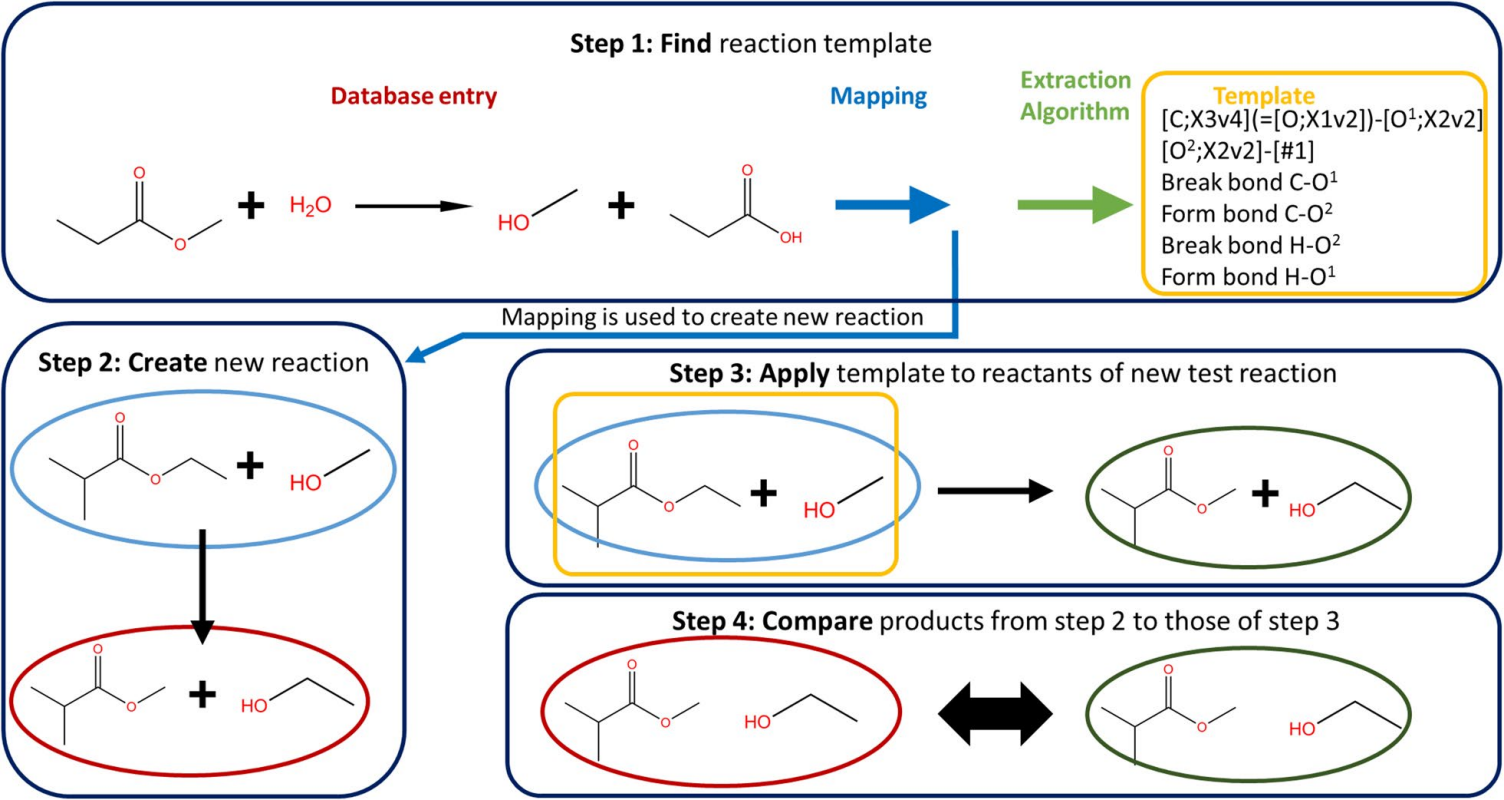
Illustration of the concept of test reactions. A database entry is analyzed and a reaction template is extracted using the described algorithm. A second step is generating a new “test” reaction, based on the available mapping. The generated template is applied to the test reaction via Genesys. In the fourth step, the generated products are compared to the expected products of the test reaction as constructed in step 2

The method is tested for each reaction as follows. The corresponding template is extracted, whereafter a set of 25 test reactions is generated. These test reactions are generated using the database entry from which the template was extracted and the available mapping for that entry. Random fragments are added and removed from reactants and products such that in the end a different reaction is obtained that has the same reactive center. The template is used in Genesys to generate a network. If a correct template was created, the products of the test reaction should be found in the species of the generated network. This method is schematically represented by Fig. 13. It should be noted that when calculated mappings are used, the accuracy of the test relies on the accuracy of the mapping. Incorrect mappings can result in false positive test outcomes.

**Fig. 14:**
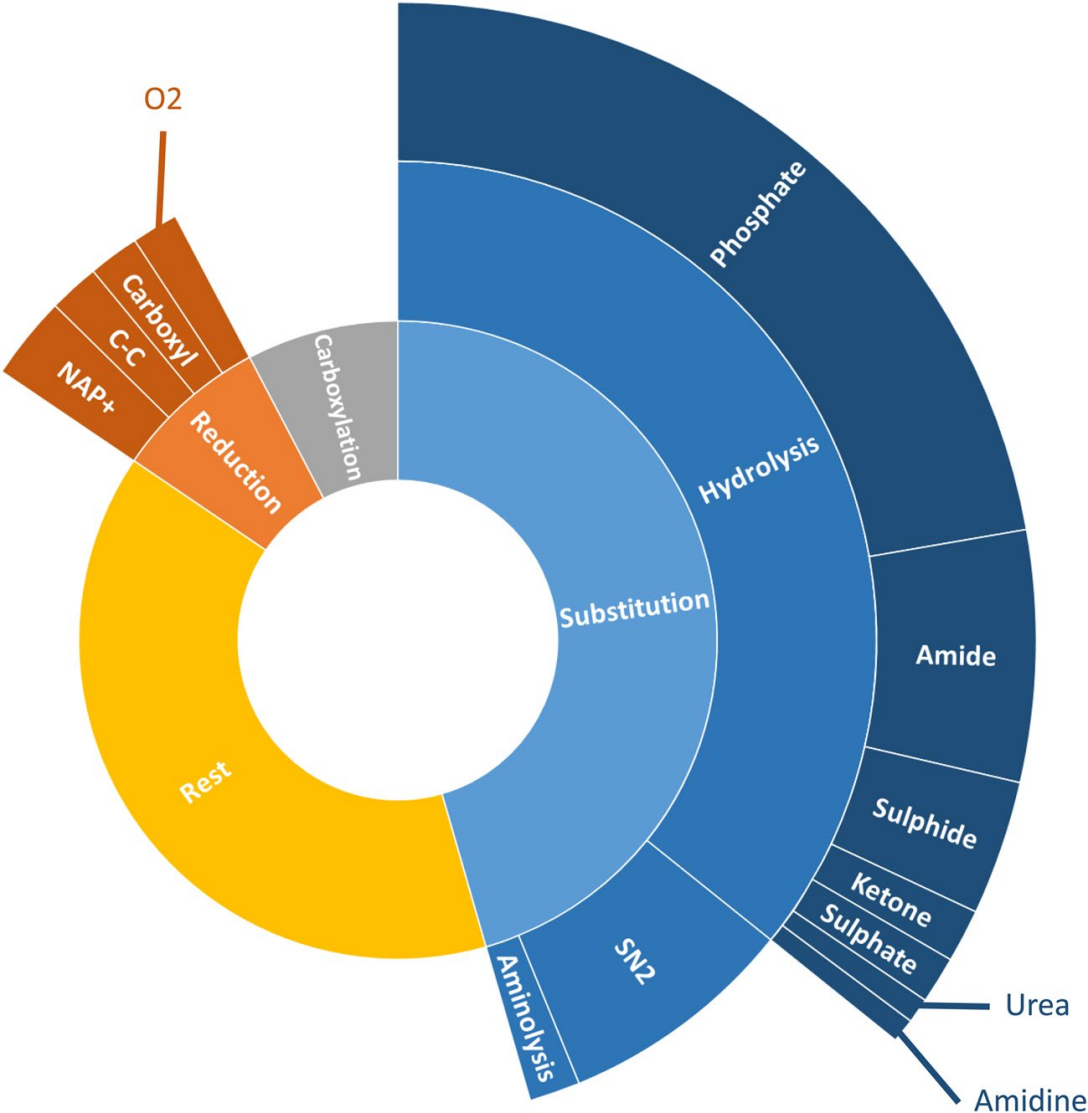
Overview of the types of templates extracted from entries R00002–R01500 of the KEGG database. Rings farther from the center group increasingly specific reaction templates

**Fig. 15:**
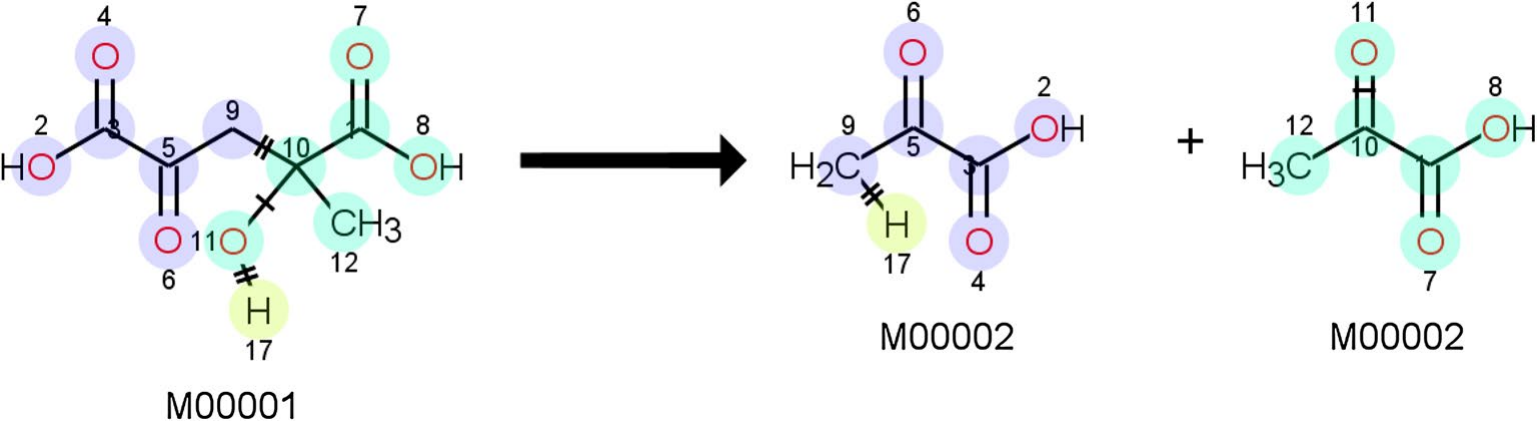
KEGG entry R00008. Example of a carbon–carbon bond forming reaction

The analyzed set results in the extraction of 238 reaction templates. An overview of all templates can be found in S-1.2. This drops to 185 when the option to include nearest neighbor heteroatoms is switched off. Figure 14 shows how the reactions are distributed across the different templates. 45% of the reactions in the KEGG subset are substitutions of which most can be classified as hydrolysis reactions. This does not imply that the reaction templates with fewer representatives are less important. In case of retro-synthetic analysis, the goal is to arrive to simpler molecules. A substitution of a functional group can be useful in some cases, but limit the amount of simplification possible. Therefore, it is necessary to include templates such as the one extracted from entry R00008 in Fig. 15, that allow carbon–carbon bond formations. These templates with a limited number of representing reactions are those that will most likely be overlooked in case of manual construction and enumeration, demonstrating the necessity of automatically extracting templates from a database in the context of retro-synthesis. The validity of the templates is not limited to the cases encountered in the database, as they can be applied to any reactant that matches the template criteria.

No cases were labeled as failures, though 28 reactions were labeled as identical. All 28 reactions were isomerizations in which the only detected change is a change in R–S or E–Z stereochemistry. Though the change is detected and stored throughout the extraction. It is lost in the formalization block, as there is no corresponding change in Genesys. As a result the reaction template for Genesys will present no net changes and hence is labeled as identical. Every single extracted reaction template was found to be compatible with Genesys.

### RMG

Three sub-databases of the RMG kinetics library, totaling 820 reactions, are analyzed using the method as described in the previous paragraph to compare the performance on organic reactions to that on pyrolysis and combustion reactions. For the same reason as given previously, reactions with three or more reactants have been omitted. The “C3” and “vinylCPD_H” databases are fractions of a larger network developed for the pyrolysis of cyclopentadiene [45]. The “Dooley/methylformate” contains information of reactions related to the combustion/pyrolysis of methylformate [46]. Further information on the data is provided in S-2.1.

**Fig. 16:**
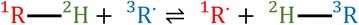
Definition of the H-Abstraction reaction template in RMG [47]. R indicates any side chain

In total 238 reaction templates are extracted, which corresponds to a similar ratio of reaction templates to reactions from which they are extracted as for the KEGG database, but is much higher than the 46 reaction templates that are used by RMG [47] to describe pyrolysis and oxidation reactions. Detailed information on the templates can be found in S-2.2. Although there is a large difference in number of reaction templates, it should be noted that the extracted reaction templates are much more specific. For example, the template “H_Abstraction” in Fig. 16 gives a large number of possible combinations when specifying the ‘R’ groups as is done here. This results in different reaction templates for hydrogen abstraction from a carbon atom by a carbon centered radical, from a carbon atom by a hydrogen centered radical, … When oxygen is also present, this results in 9 different reaction templates. As most reaction template definitions in RMG contain at least 2 ‘R’ groups, an extrapolation of a one-to-nine ratio of reaction templates implies that around 30 of the reaction templates defined in RMG are retrieved from the investigated databases.

4 reactions were labeled as identical. In these cases, part of the reaction describes the transformation of the CH singlet state to the CH triplet state. At the moment, it is not possible to transfer this information via standardized molecular identifiers, resulting in the two states being considered identical.

**Fig. 17:**
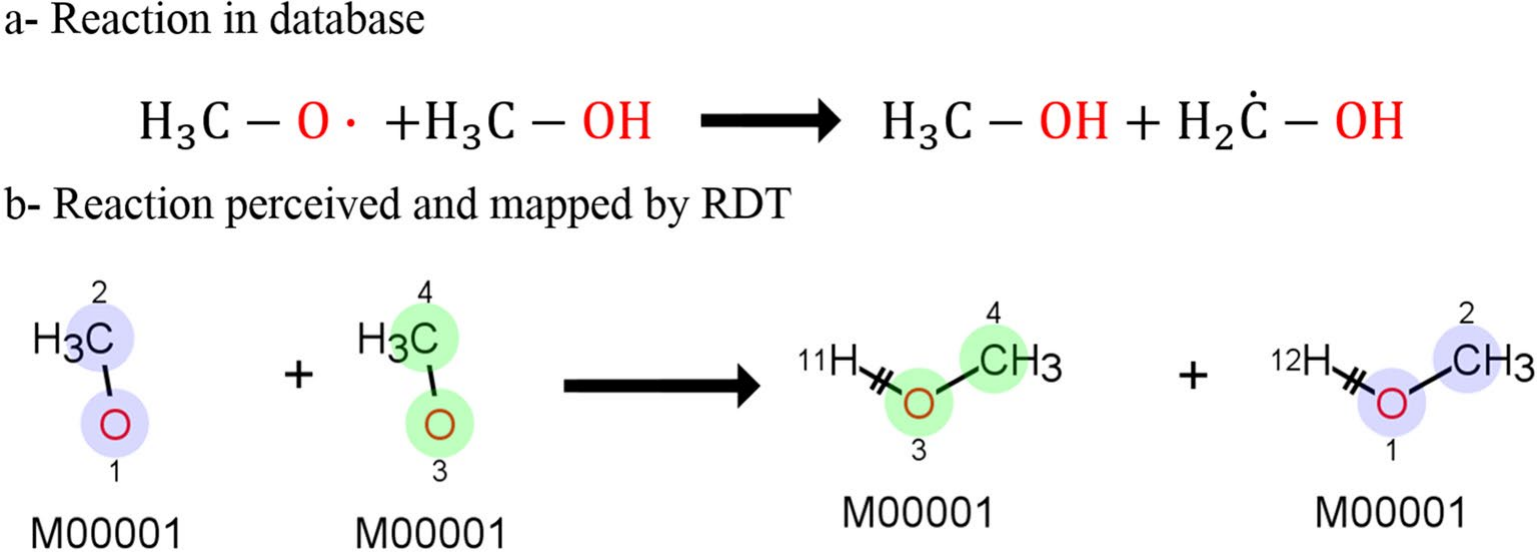
Misinterpretation of radical species in the RDT. Reactants and products are compared separately. Fingerprints cannot distinguish radicals, so the second reactant is perceived to be equal to the first. The same happens for products

11 reactions were labeled as failures by the algorithm. The two sources of error in the algorithm are the AAM, which is colored blue in Fig. 5 and the extraction of the reactive center, which is green in Fig. 5. Reactions for which the AAM fails to generate a complete mapping, are not analyzed further. This is the case for 2 of the 820 reactions, or 0.25%. This success rate is close to the one reported for the RDT [37], showing the flexibility of the slightly adapted RDT to handle pyrolysis reactions as well as organic reactions. Besides improving the handling of radicals by the RDT, a second important adaption was using InChIs to distinguish between molecules. The initial use of molecular fingerprints [48] works well for organic molecules, but fails for radicals. While being a fast method to compare molecules, their definition makes it impossible to distinguish between a given species and a radical derived from it, *e.g.* methane and the methyl radical. Therefore certain reactions, such as the example in Fig. 17, were identified as identical. Fingerprints are still used as identifier in those cases for which an InChI cannot be determined. For nine other cases or 1.1%, a second type of failure is reported. In these cases the failure is issued by the “mechanism acceptable” block in Fig. 5. In all of them, the criteria that if radicals are present in the reactants, they should participate in the mechanism, was not met. Analysis of the reactions indicate that in some of those cases the calculated mappings are—at least—plausible, i.e. it is possible that on a net base, the radicals do not participate. The testing method described at the beginning of the section showed that all 238 reaction templates resulted in the correct products being formed.

### Comparison of a 1,5-hexadiene and a methyl butanoate model

A final test of the algorithm is the analysis and comparison of published reaction networks. In the ideal case, for each of the networks, the exact same reaction templates will be identified as those from which the network was generated. On the one hand, a reaction network for the pyrolysis and oxidation of 1,5-hexadiene [49] comprising 8610 reactions is analyzed. On the other, a reaction network for the pyrolysis of methyl butanoate [50] comprising 20,220 reactions is analyzed. Both reaction networks are provided in S-3.1. The analysis of the hexadiene network took approximately 13 h on an Intel i7-6820HQ 2.7 GHz processor, which averages to 5.4 s per reaction. Over 95% of the computational time is spent on generating the AAM, with total analysis time dropping to only 0.21 h or 0.1 s per reaction, if predetermined mappings are used. For the methyl butanoate network, this is 2.8 s and 0.22 s respectively, which indicates that less time is being spent on calculating the mappings.

**Fig. 18:**
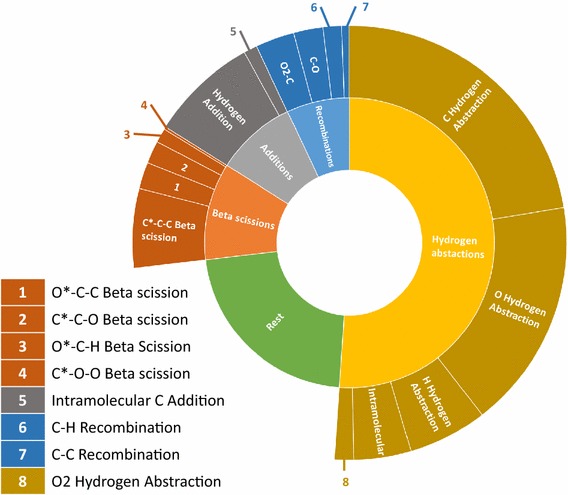
Distribution of the reactions in the hexadiene model. The outer ring makes an additional specification of the groups

**Fig. 19:**
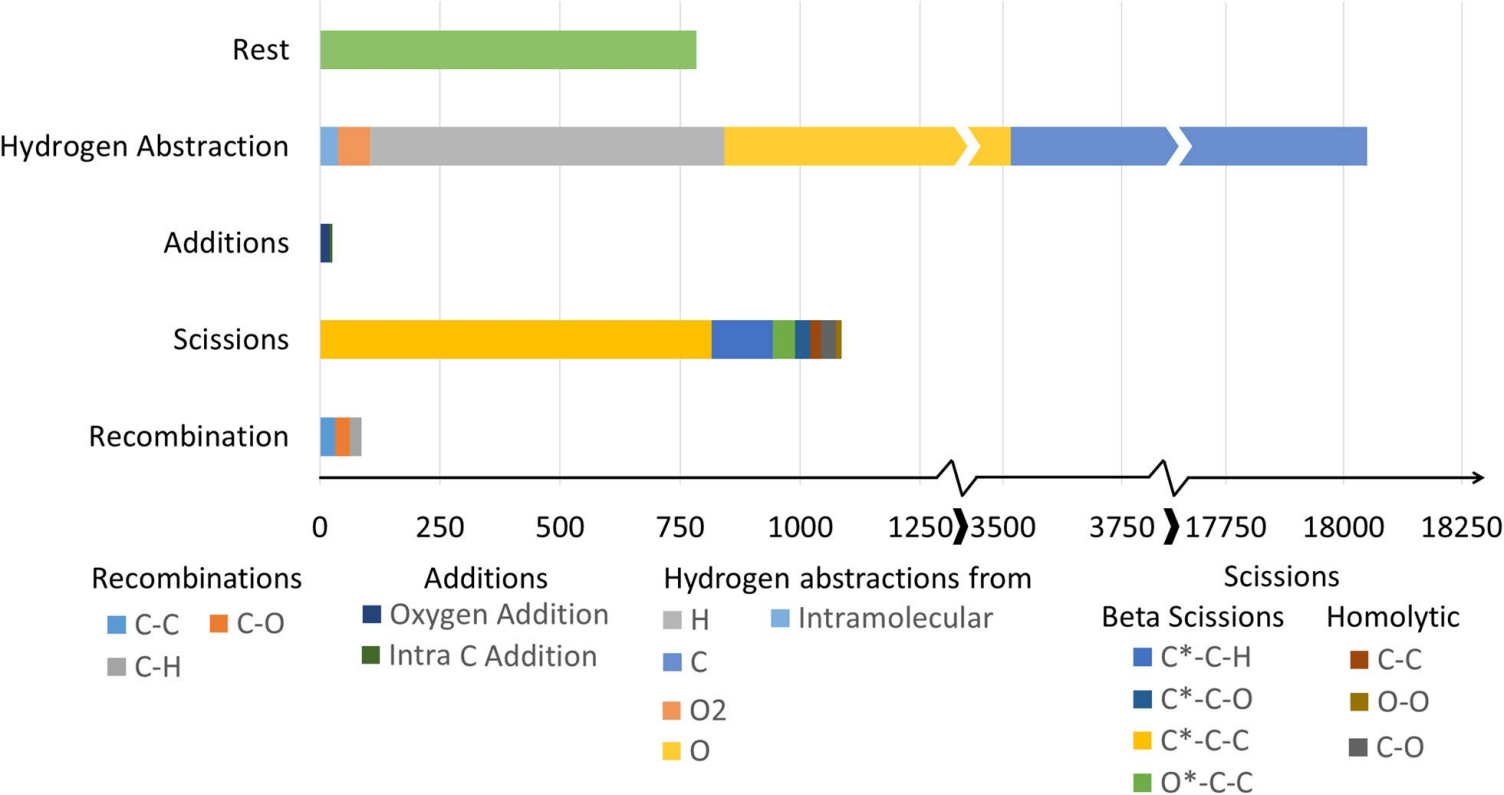
Distribution of the reactions in the methyl butanoate model

Of the 8610 hexadiene reactions, 49 reactions, or 0.6%, could not be assigned an AAM or the determined mapping was construed as incorrect. A total of 803 reaction templates are extracted from the network. Thereof 296 are the reverse of another reaction template. It is correct for the number of reverse templates to be slightly less than half of the total number of templates, as for some reactions, both the forwards and the reverse path follow the same template. The methyl butanoate network contains significantly more reactions, but these are covered by just over half the number of templates: 476, of which 215 are labeled ‘reverse’. A detailed overview of the extracted templates can be found in S-3.2. One of the reasons for the large difference is the inclusion of aromatics chemistry in the hexadiene network. A significant portion of the aromatics chemistry is included via base-mechanisms, which include well-studied reactions [51, 52]. Many of these reactions are intra-molecular reactions and involve a variety of ring structures. These rings can be of various size and contain several bond types or elements. Each different ring element, size or bond type will demand a separate template, greatly increasing the number of extracted templates. In analogy to the previous paragraph, Fig. 18 shows an analysis of how many reactions in the network represent each reaction template for the hexadiene model. The data for the methyl butanoate model is displayed in Fig. 19, using the same general reaction classes. In both cases, the vast majority of reactions can be categorized into 5 major classes and around 16 sub-classes. Some of these subclasses contain more than one template, for example the group “carbon centered hydrogen abstraction” groups the templates that describe abstraction of hydrogen by a carbon atom from a carbon, oxygen or hydrogen atom. In both cases, there is a clear dominance of the templates describing hydrogen abstractions. In the hexadiene network 47% of the analyzed reactions can be categorized as hydrogen abstraction reactions, while in the methyl butanoate network this is 90%. Other important reaction classes in the hexadiene network, covering about 30% of the reactions in the hexadiene network and 6% in the methyl butanoate network, are hydrogen shifts, radical recombinations, additions and beta scissions. A significant difference is observed between the number of representing reactions for these templates in the respective networks. While beta scissions are quite well-represented in both models, there is a remarkable lack of representation of the intramolecular hydrogen abstractions, recombinations and addition reactions in the methyl butanoate network. The corresponding reactions belong to the section of the model that was automatically generated by Genesys. Therefore, they are the result of manually constructed and constrained templates. The templates for the intramolecular hydrogen abstractions use very strict constraints. Additionally, the methyl butanoate model focusses on species with five or fewer carbon atoms, limiting the number of possible intra-molecular hydrogen abstractions. The addition and recombination templates limit the number of atoms allowed in the reagents. This reflects the nature of both systems. Due to the heavier starting molecule and lack of oxygen, the pyrolysis of hexadiene will typically result in important chain growth and aromatics formation. This is implies accounting for a large number of addition and recombination reactions. Methyl butanoate pyrolysis is a very different system as the starting molecule introduces oxygen into the system. The resulting oxidation reactions favor the formation of shorter chains and CO/CO_2_, making it less important to account for chain growth reactions.

Except for the templates in the 16 groups of Fig. 18, the majority has a very low number of representing reactions. The limited number of reactions not covered by one of the 16 major templates follow relatively unique templates, which is in accordance with the practice of using well-known and highly specific base mechanisms that give rise to a wide variety of templates. The total of 108 reaction templates that were encoded for the automatic generation of the hexadiene mechanism cover the same reactions as the 16 major templates and some of the more unique templates, but fail to describe around 700 templates in the rest group. This again demonstrates the importance of automatically extracting templates, either from existing networks or databases if fully automated reaction network generation is aimed for.

A second analysis is performed for the hexadiene model, in which the base mechanisms are left out. This results in a network that is based on just the 108 coded reaction templates. From that network, 97 reaction templates are extracted. Admittedly, this does not exactly equal the number of encoded reaction templates. However, several of the encoded reaction templates describe the same transformation, but are assigned different constraints and kinetics, resulting in them being defined separately. These nuances are not captured by the reaction analysis algorithm. A closer look at the encoded reaction templates learns that only 88 different transformations are uniquely described. Uniquely here means being identical after removing all constraints and additional information, retaining only the essential description of the transformation. The remaining difference arises from differences in the specificity of the reaction templates. For example, the algorithm finds two types of hydrogen abstractions from a carbon atom by a carbon radical. In one, no heteroatoms are bonded to the non-radical carbon, while in the other the non-radical carbon has an oxygen as nearest neighbor. Investigation of the described chemistry by the encoded reaction templates and the extracted reaction templates learns that in the end, they describe the same chemistry. This shows that the automatically extracted templates cover the exact same chemistry as was intended by the original user and demonstrates the reliability of the tool.

## Conclusions

An algorithm has been developed to automate the generation of reaction templates from databases. Depending on which database is used, the templates can be used for the prediction of retro-synthetic steps or as input for kinetic model generation tools, such as Genesys. The algorithm comes with tools to interpret several different type of chemical database formats such as CHEMKIN^®^ input and the KEGG database. If necessary, missing AAMs are completed using the RDT. This is a very time consuming step: up to 94% of the computing time is spent on calculating the AAM. Any improvements in this field will greatly speed up the entire process. From the AAM, the changes and reactive atoms are extracted resulting in a reaction template. Given a correct AAM, flawless generation of the template is possible, both for organic, non-elementary reactions as for pyrolysis/combustion, elementary reactions. The comparison of two different kinetic models shows very similar templates are extracted from networks describing very different systems. The number of reactions representing each of those templates varies strongly and can form an indication of the types of chemistry that are important in each system. Future developments may allow for simultaneous extraction of kinetics and kinetic parameters derived thereof. The large amounts of kinetic data included in a CHEMKIN^®^ network could make it possible to derive group additive values for reaction templates of which a large number of members are present in the analyzed network.

The algorithm described here brings us one step closer to fully automating the generation of detailed and accurate kinetic models. It eliminates the time consuming step of defining the necessary reaction templates and provides opportunities to further facilitate the usage of the extensive chemical knowledge that is present in chemical databases, for example in retro-synthetic analysis of drug syntheses.

## Additional files

**Additional file 1.** Input file for analysis of the hexadiene network.

**Additional file 2.** Input file for analysis of the methyl butanoate network.

**Additional file 3.** Documentation of the supporting information.

**Additional file 4.** Reaction template output for the hexadiene network.

**Additional file 5.** Reaction template output for the methyl butanoate network.

**Additional file 6.** Reaction template output for the RMG database analysis.

**Additional file 7.** Reaction template output for the KEGG database analysis.

**Additional file 8.** Zip-file containing all necessary data/files to run the software.

**Additional file 9.** Zip-file containing the source code of the software.

## Abbreviations

RMG: reaction mechanism generator
InChI: international chemical identifier
SMILES: simplified molecular input line system
SMARTS: SMILES arbitrary target specification
KEGG: Kyoto encyclopedia of genes and genomes
AAM: atom–atom mapping
CDK: chemical development kit
JNI: Java native interface
MDL: Molecular Design Limited
GUI: graphical user interface
RDT: reaction decoder tool
MCS: maximum common substructure
GAV: group additive value
S: supporting information
C_1_: hydrocarbons containing 1 carbon atom
C_2_: hydrocarbons containing 2 carbon atoms
r: molecular radius with respect to a certain atom
i: counting index
