# Supplementary material for: Automated reaction database and reaction network analysis: extraction of reaction templates using cheminformatics
Source: J Cheminform. 2018 Mar 9;10:11. doi: 10.1186/s13321-018-0269-8 (PMC5845084; doi:10.1186/s13321-018-0269-8)
Supplement: Supplementary file 3 — Additional file 3. Documentation of the supporting information. [file 13321_2018_269_MOESM3_ESM.docx]

**Automated Reaction Database and Reaction Network Analysis: Extraction of Reaction Templates using Cheminformatics**

**Supporting Information**

Pieter P. Plehiers^1^, Guy B. Marin^1^, Christian V. Stevens^2^, Kevin M. Van Geem^1,*^

^1^Laboratory for Chemical Technology, Department of Materials, Textiles and Chemical Engineering, Ghent University, Technologiepark 914 9052 Gent, Belgium

^2^SynBioC Research Group, Department of Sustainable Organic Chemistry and Technology,

Faculty of Bioscience Engineering, Ghent University, Coupure Links 653, 9000 Gent, Belgium

^*^ Corresponding author: [Kevin.VanGeem@UGent.be](mailto:Kevin.VanGeem@UGent.be), Technologiepark 914, 9052 Gent, Belgium;

Contents

[1. Supporting Information to section 3.1 3](#_Toc499804352)

[1.1. Used KEGG Reaction IDs 3](#_Toc499804353)

[1.2. Extracted Templates 6](#_Toc499804354)

[2. Supporting Information to section 3.2 7](#_Toc499804355)

[2.1. Used RMG reactions 7](#_Toc499804356)

[2.2. Extracted Templates 7](#_Toc499804357)

[3. Supporting Information to section 3.3 7](#_Toc499804358)

[3.1. Chemkin^®^ Reaction Networks 7](#_Toc499804359)

[3.2. Extracted Templates 7](#_Toc499804360)

[4. Source Code and Executable 7](#_Toc499804361)

# Supporting Information to section 3.1

## Used KEGG Reaction IDs

The following list provides all KEGG reaction ids of the reactions that were used for evaluation in section 3.1. Reactions with three or more reagents have already been left out. Further information on each entry can be accessed via [www.kegg.jp/entry/[reaction](http://www.kegg.jp/entry/%5breaction) id]. The molecular data files of products and reactants can be accessed by continuing to the respective pages of each molecule.

R00002, R00004, R00005, R00006, R00008, R00009, R00011, R00012, R00013, R00014, R00015, R00018, R00019, R00021, R00022, R00026, R00027, R00028, R00032, R00033, R00036, R00038, R00039, R00043, R00048, R00049, R00053, R00054, R00056, R00059, R00062, R00063, R00066, R00067, R00069, R00086, R00087, R00088, R00089, R00100, R00101, R00104, R00105, R00106, R00108, R00109, R00112, R00118, R00119, R00122, R00123, R00125, R00126, R00127, R00128, R00129, R00130, R00131, R00132, R00134, R00138, R00139, R00140, R00141, R00155, R00156, R00157, R00158, R00159, R00160, R00161, R00173, R00174, R00175, R00178, R00179, R00180, R00181, R00182, R00183, R00184, R00185, R00187, R00188, R00190, R00191, R00192, R00193, R00194, R00195, R00198, R00200, R00208, R00212, R00213, R00214, R00215, R00216, R00217, R00218, R00219, R00220, R00221, R00223, R00224, R00226, R00227, R00230, R00233, R00236, R00237, R00238, R00239, R00241, R00256, R00258, R00259, R00260, R00261, R00262, R00267, R00268, R00269, R00270, R00272, R00273, R00276, R00278, R00287, R00289, R00291, R00293, R00299, R00300, R00301, R00302, R00303, R00304, R00305, R00306, R00307, R00310, R00315, R00316, R00317, R00318, R00319, R00320, R00321, R00322, R00324, R00325, R00327, R00328, R00330, R00332, R00334, R00335, R00336, R00337, R00338, R00339, R00340, R00341, R00342, R00343, R00345, R00346, R00347, R00348, R00350, R00351, R00353, R00354, R00355, R00360, R00361, R00362, R00363, R00367, R00369, R00371, R00372, R00373, R00391, R00395, R00397, R00399, R00400, R00401, R00402, R00406, R00407, R00408, R00409, R00410, R00411, R00414, R00416, R00417, R00418, R00420, R00424, R00426, R00428, R00429, R00430, R00431, R00434, R00445, R00449, R00451, R00453, R00454, R00457, R00458, R00460, R00461, R00462, R00463, R00465, R00469, R00470, R00471, R00472, R00473, R00474, R00475, R00477, R00478, R00479, R00480, R00481, R00484, R00485, R00487, R00488, R00489, R00490, R00491, R00493, R00494, R00499, R00502, R00503, R00504, R00505, R00507, R00508, R00509, R00510, R00511, R00512, R00513, R00514, R00515, R00516, R00517, R00518, R00519, R00521, R00522, R00524, R00525, R00526, R00527, R00529, R00530, R00531, R00532, R00534, R00535, R00536, R00537, R00548, R00549, R00550, R00551, R00552, R00554, R00559, R00565, R00566, R00567, R00568, R00569, R00570, R00572, R00574, R00576, R00577, R00579, R00582, R00584, R00585, R00586, R00588, R00589, R00590, R00594, R00595, R00596, R00597, R00598, R00599, R00600, R00601, R00602, R00603, R00605, R00606, R00608, R00615, R00616, R00617, R00618, R00619, R00621, R00623, R00624, R00625, R00626, R00627, R00628, R00629, R00636, R00637, R00643, R00644, R00647, R00650, R00651, R00652, R00653, R00654, R00655, R00656, R00658, R00659, R00660, R00661, R00662, R00665, R00667, R00668, R00669, R00670, R00671, R00672, R00673, R00674, R00678, R00679, R00681, R00682, R00683, R00684, R00685, R00690, R00691, R00692, R00693, R00694, R00695, R00697, R00699, R00700, R00702, R00703, R00704, R00709, R00717, R00719, R00720, R00722, R00723, R00724, R00725, R00726, R00728, R00732, R00733, R00734, R00736, R00737, R00739, R00743, R00746, R00747, R00748, R00749, R00750, R00751, R00753, R00754, R00755, R00756, R00758, R00760, R00761, R00762, R00763, R00764, R00765, R00766, R00767, R00768, R00769, R00770, R00771, R00772, R00775, R00776, R00777, R00778, R00782, R00793, R00801, R00802, R00803, R00805, R00806, R00807, R00809, R00810, R00811, R00812, R00813, R00814, R00816, R00817, R00821, R00822, R00827, R00829, R00830, R00832, R00833, R00834, R00835, R00836, R00837, R00838, R00839, R00841, R00842, R00844, R00845, R00846, R00847, R00848, R00849, R00850, R00851, R00853, R00854, R00855, R00856, R00857, R00863, R00866, R00867, R00868, R00870, R00871, R00872, R00874, R00875, R00876, R00877, R00878, R00881, R00883, R00885, R00888, R00889, R00891, R00893, R00895, R00896, R00897, R00899, R00901, R00903, R00905, R00907, R00908, R00909, R00911, R00913, R00917, R00919, R00921, R00923, R00924, R00926, R00927, R00928, R00930, R00931, R00932, R00934, R00936, R00939, R00944, R00946, R00947, R00948, R00949, R00951, R00952, R00953, R00954, R00955, R00956, R00959, R00960, R00961, R00962, R00963, R00964, R00965, R00966, R00967, R00968, R00969, R00970, R00973, R00974, R00975, R00977, R00978, R00983, R00984, R00985, R00986, R00987, R00988, R00989, R00990, R00995, R00996, R00997, R00998, R00999, R01000, R01001, R01003, R01004, R01005, R01006, R01007, R01008, R01009, R01010, R01011, R01012, R01013, R01014, R01015, R01016, R01018, R01021, R01022, R01023, R01026, R01030, R01031, R01034, R01036, R01039, R01041, R01043, R01044, R01047, R01049, R01050, R01051, R01052, R01054, R01055, R01056, R01057, R01059, R01064, R01066, R01067, R01068, R01069, R01070, R01071, R01073, R01074, R01076, R01077, R01080, R01081, R01082, R01083, R01085, R01086, R01087, R01089, R01090, R01091, R01092, R01094, R01096, R01097, R01099, R01100, R01101, R01103, R01104, R01111, R01117, R01121, R01123, R01126, R01127, R01128, R01131, R01132, R01137, R01138, R01139, R01140, R01144, R01145, R01146, R01147, R01148, R01149, R01152, R01153, R01154, R01155, R01156, R01157, R01159, R01160, R01161, R01166, R01167, R01168, R01171, R01174, R01175, R01177, R01179, R01180, R01181, R01183, R01184, R01185, R01186, R01187, R01188, R01189, R01190, R01192, R01193, R01194, R01195, R01199, R01200, R01201, R01204, R01205, R01207, R01209, R01213, R01214, R01215, R01216, R01218, R01220, R01224, R01227, R01228, R01229, R01232, R01233, R01234, R01235, R01236, R01238, R01239, R01240, R01241, R01242, R01243, R01244, R01245, R01246, R01248, R01249, R01251, R01253, R01255, R01257, R01261, R01262, R01265, R01266, R01267, R01268, R01270, R01271, R01274, R01278, R01279, R01281, R01283, R01286, R01287, R01288, R01289, R01290, R01291, R01293, R01301, R01302, R01304, R01305, R01307, R01313, R01314, R01320, R01321, R01323, R01324, R01325, R01326, R01327, R01328, R01329, R01330, R01334, R01335, R01336, R01341, R01344, R01346, R01349, R01351, R01352, R01353, R01354, R01355, R01358, R01359, R01360, R01361, R01364, R01365, R01366, R01367, R01368, R01370, R01371, R01372, R01373, R01375, R01376, R01377, R01378, R01381, R01383, R01384, R01385, R01386, R01387, R01388, R01390, R01392, R01393, R01394, R01395, R01396, R01397, R01398, R01399, R01401, R01402, R01403, R01404, R01408, R01411, R01414, R01415, R01416, R01417, R01418, R01421, R01423, R01424, R01425, R01429, R01430, R01431, R01432, R01437, R01440, R01441, R01442, R01444, R01445, R01447, R01448, R01449, R01450, R01451, R01456, R01457, R01459, R01460, R01461, R01462, R01465, R01466, R01467, R01468, R01470, R01471, R01472, R01473, R01476, R01478, R01480, R01481, R01482, R01483, R01484, R01485, R01487, R01488, R01491, R01494, R01495, R01496, R01497, R01498, R01500

## Extracted Templates

For a full overview of the extracted templates from the reactions mentioned above, the file titled “reaction_families_KEGG.xml” is provided along with this supporting information.

# Supporting Information to section 3.2

## Used RMG reactions

All reactions in the online RMG databases named “C3”, “Dooley/methylformate” and “vinylCPD_H” were used for the evaluation in section 3.2. The databases can be accessed online via [http://rmg.mit.edu/database/kinetics/libraries/[database](http://rmg.mit.edu/database/kinetics/libraries/%5bdatabase) name].

## Extracted Templates

For a full overview of the extracted templates from the reactions found in the databases mentioned above, the file titled “reaction_families_RMG.xml” is provided along with this supporting information.

# Supporting Information to section 3.3

## Chemkin^®^ Reaction Networks

The input format used for the testing in section 3.3 was a Chemkin^®^ reaction network file. The files for respectively the methylbutanoate and hexadiene networks are provided as separate files titled “methylbutanoate.inp” and hexadiene.inp”.

## Extracted Templates

Detailed information on all extracted templates for both the hexadiene and methylbutanoate networks is provided in additional files, titled “reaction_families_HEX.xml” and “reaction_families_MET.xml” respectively.

# Source Code and Executable

The full source code is provided as supporting information in “source_code.zip”. Additionally, a compiled version of the code that can be run from the command line is provided, including all other required software, such as the Reaction Decoder Tool. A demo ready-to-run setup is provided in “demo.zip”

This program is free software: you can redistribute it and/or modify

it under the terms of the GNU General Public License as published by

the Free Software Foundation, either version 3 of the License, or

(at your option) any later version.

This program is distributed in the hope that it will be useful,

but WITHOUT ANY WARRANTY; without even the implied warranty of

MERCHANTABILITY or FITNESS FOR A PARTICULAR PURPOSE. See the

GNU General Public License for more details.
