# Supplementary figures and images for: Automated reaction database and reaction network analysis: extraction of reaction templates using cheminformatics
Source: J Cheminform. 2018 Mar 9;10:11. doi: 10.1186/s13321-018-0269-8 (PMC5845084; doi:10.1186/s13321-018-0269-8)

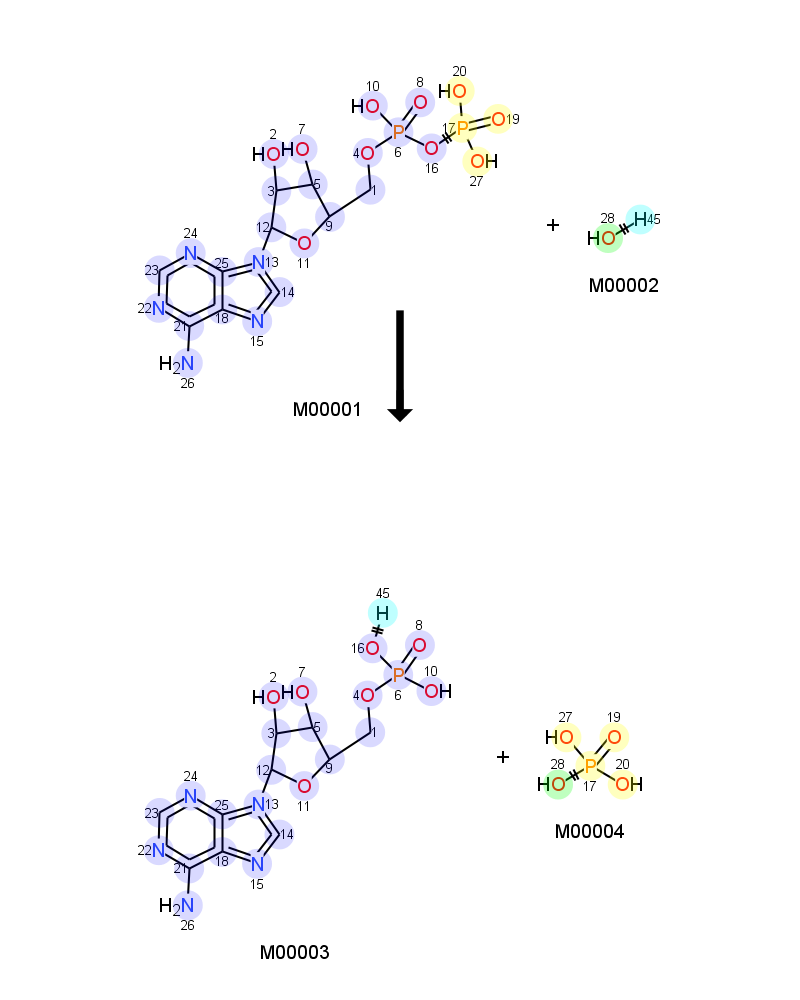

Supplement: Supplementary file 8 — Additional file 8. Zip-file containing all necessary data/files to run the software. [file 13321_2018_269_MOESM8_ESM.zip › demo/ReactionTemplates/R00001_R00122/ECBLAST_R00001_R00122_0_AAM.png]

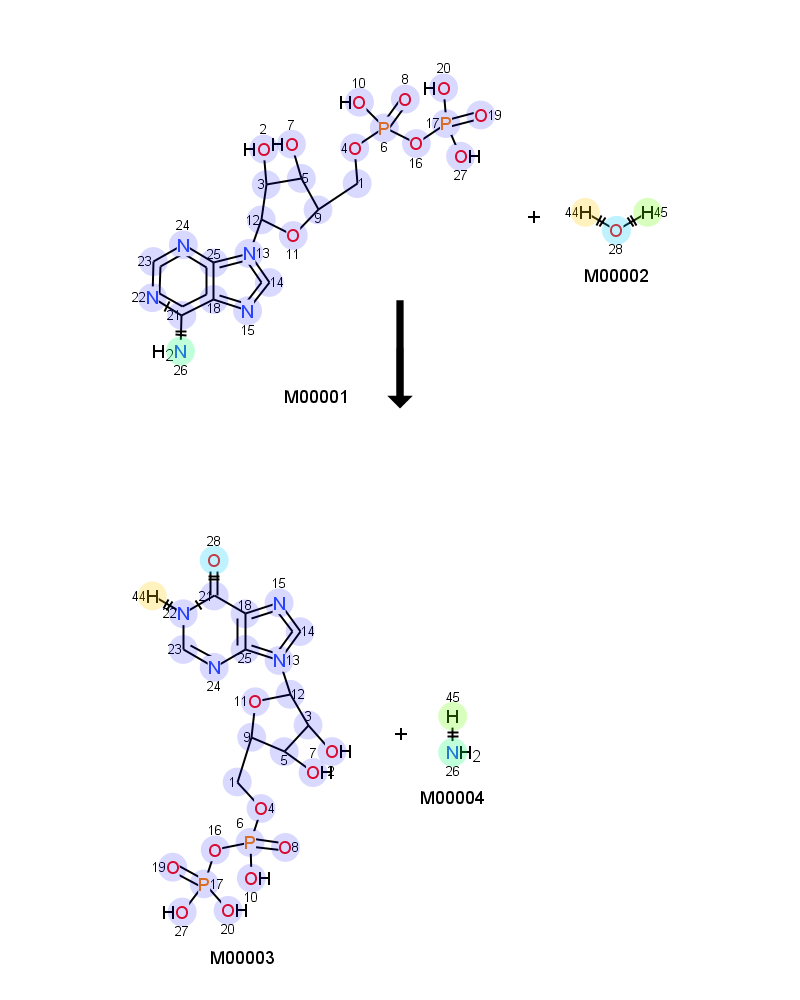

Supplement: Supplementary file 8 — Additional file 8. Zip-file containing all necessary data/files to run the software. [file 13321_2018_269_MOESM8_ESM.zip › demo/ReactionTemplates/R00002_R00123/ECBLAST_R00002_R00123_0_AAM.png]

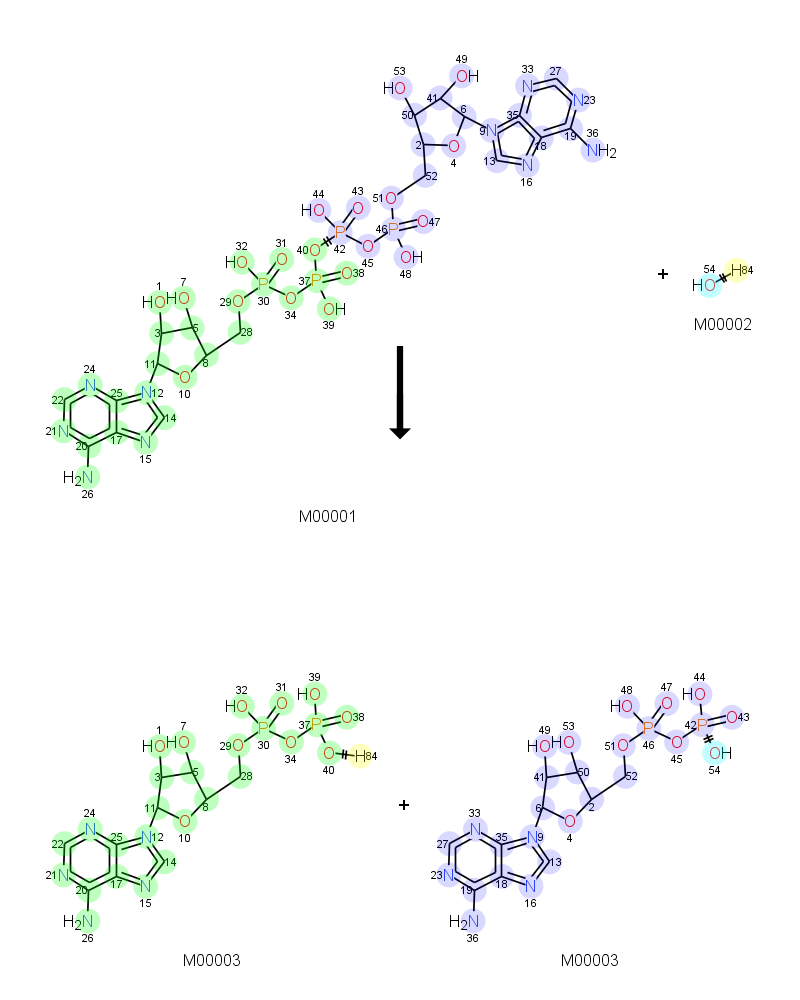

Supplement: Supplementary file 8 — Additional file 8. Zip-file containing all necessary data/files to run the software. [file 13321_2018_269_MOESM8_ESM.zip › demo/ReactionTemplates/R00003_R00125/ECBLAST_R00003_R00125_0_AAM.png]

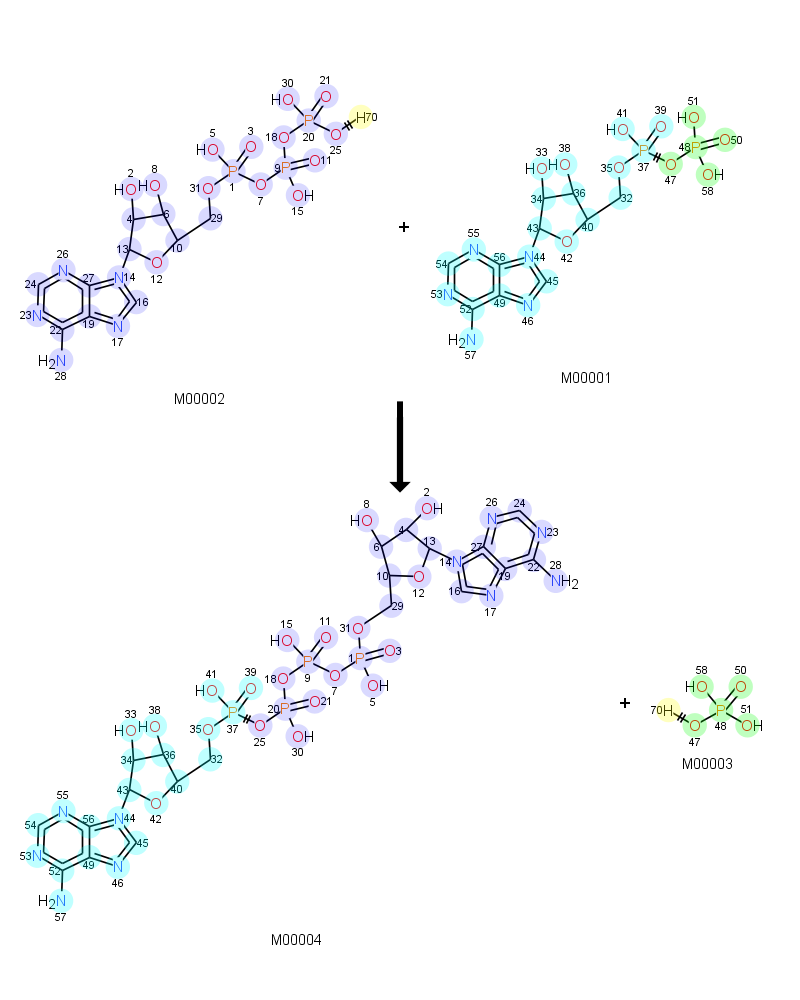

Supplement: Supplementary file 8 — Additional file 8. Zip-file containing all necessary data/files to run the software. [file 13321_2018_269_MOESM8_ESM.zip › demo/ReactionTemplates/R00004_R00126/ECBLAST_R00004_R00126_0_AAM.png]

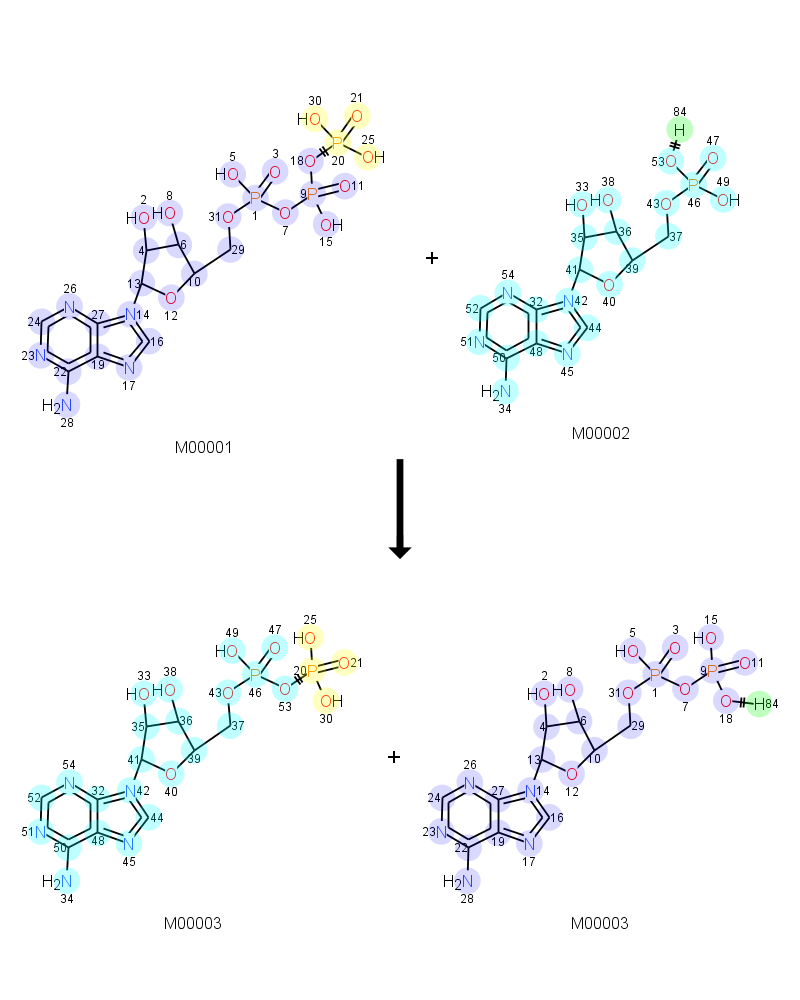

Supplement: Supplementary file 8 — Additional file 8. Zip-file containing all necessary data/files to run the software. [file 13321_2018_269_MOESM8_ESM.zip › demo/ReactionTemplates/R00005_R00127/ECBLAST_R00005_R00127_0_AAM.png]

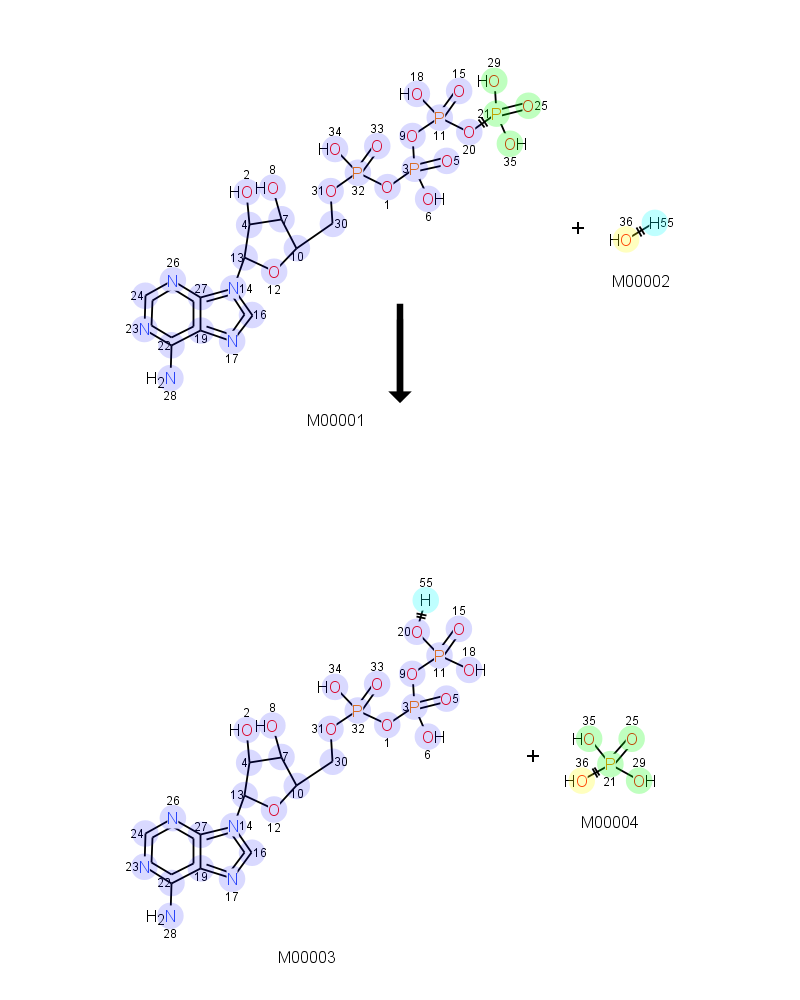

Supplement: Supplementary file 8 — Additional file 8. Zip-file containing all necessary data/files to run the software. [file 13321_2018_269_MOESM8_ESM.zip › demo/ReactionTemplates/R00006_R00128/ECBLAST_R00006_R00128_0_AAM.png]

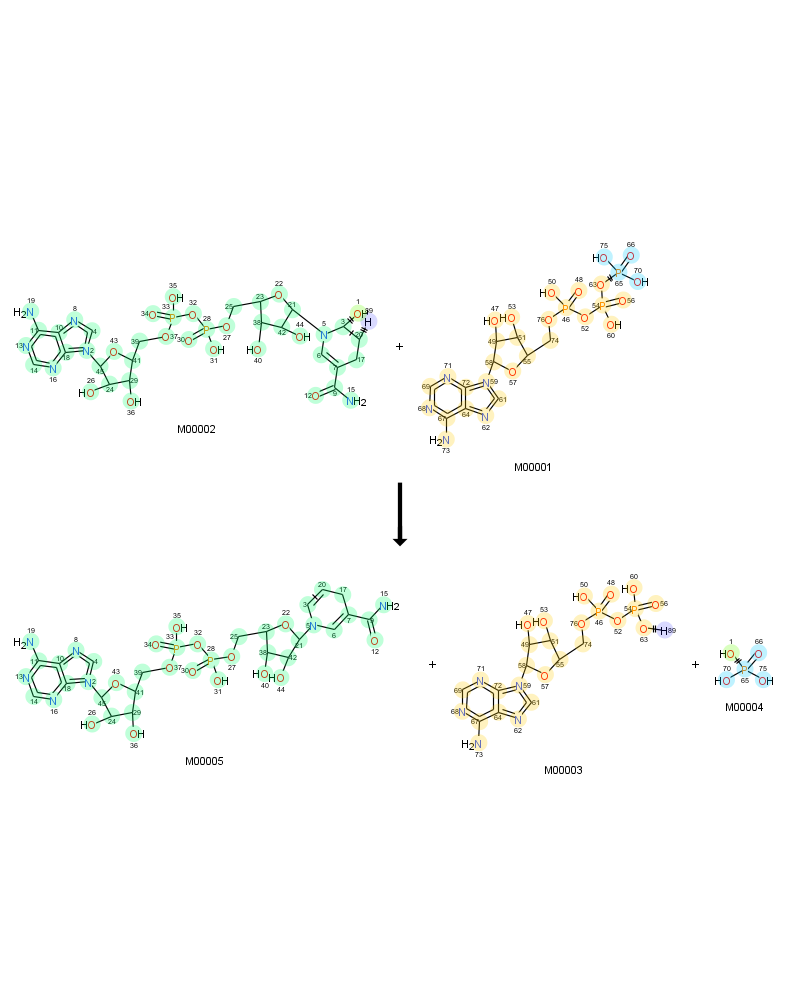

Supplement: Supplementary file 8 — Additional file 8. Zip-file containing all necessary data/files to run the software. [file 13321_2018_269_MOESM8_ESM.zip › demo/ReactionTemplates/R00007_R00129/ECBLAST_R00007_R00129_0_AAM.png]

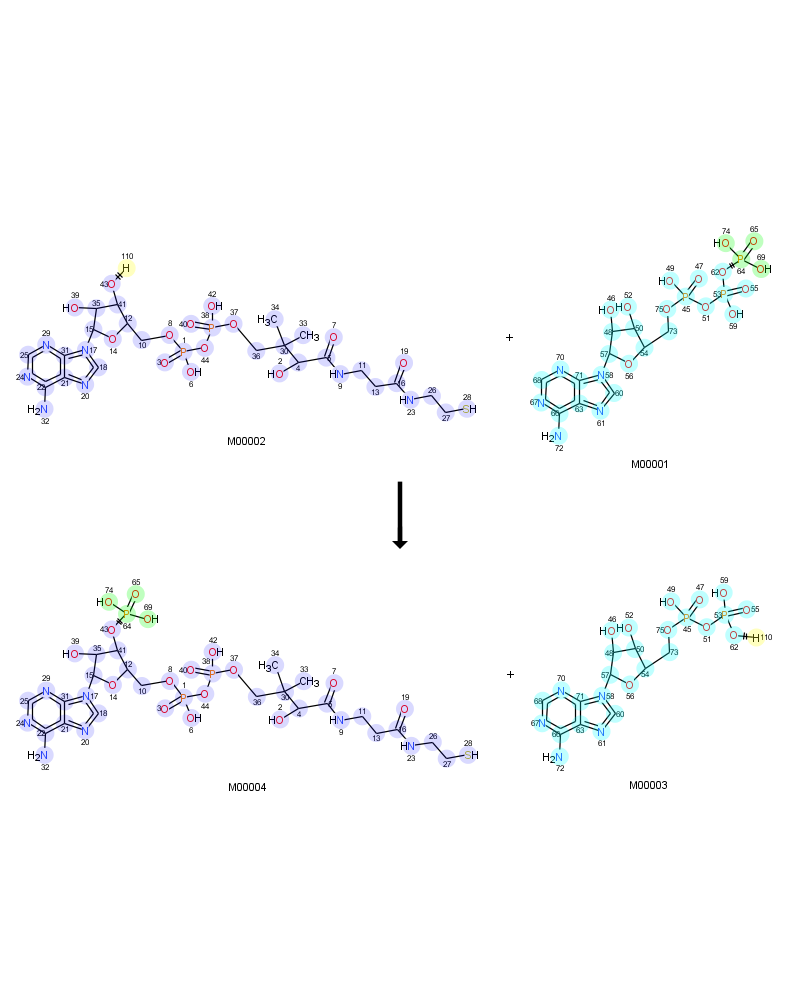

Supplement: Supplementary file 8 — Additional file 8. Zip-file containing all necessary data/files to run the software. [file 13321_2018_269_MOESM8_ESM.zip › demo/ReactionTemplates/R00008_R00130/ECBLAST_R00008_R00130_0_AAM.png]

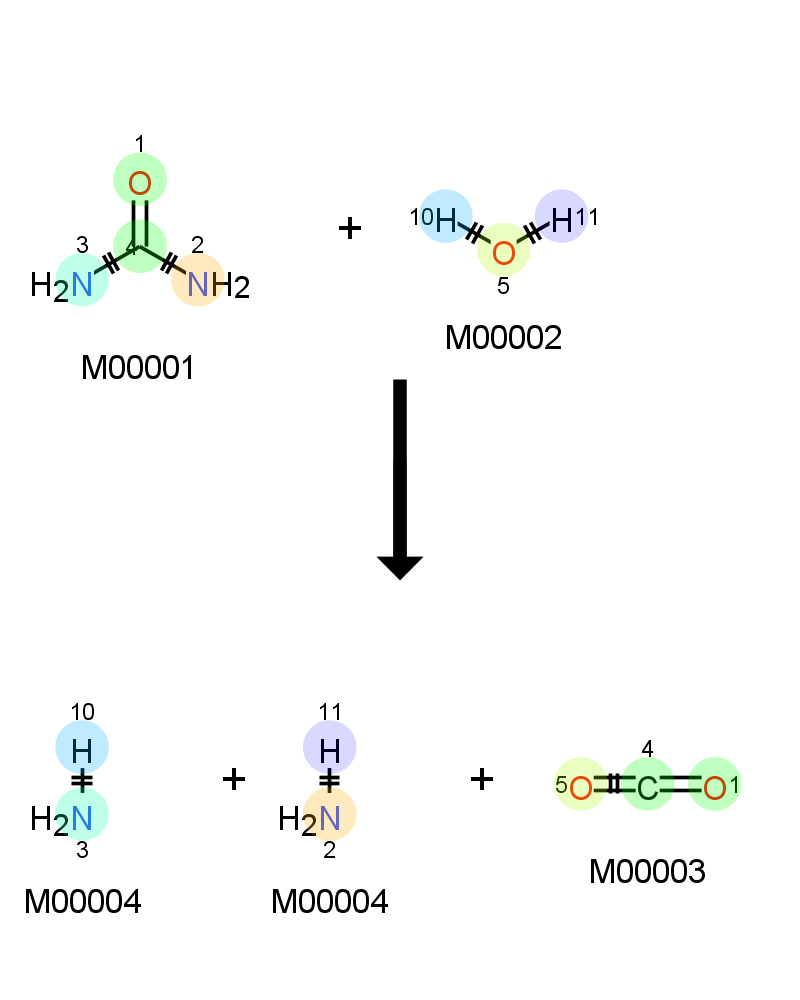

Supplement: Supplementary file 8 — Additional file 8. Zip-file containing all necessary data/files to run the software. [file 13321_2018_269_MOESM8_ESM.zip › demo/ReactionTemplates/R00009_R00131/ECBLAST_R00009_R00131_0_AAM.png]

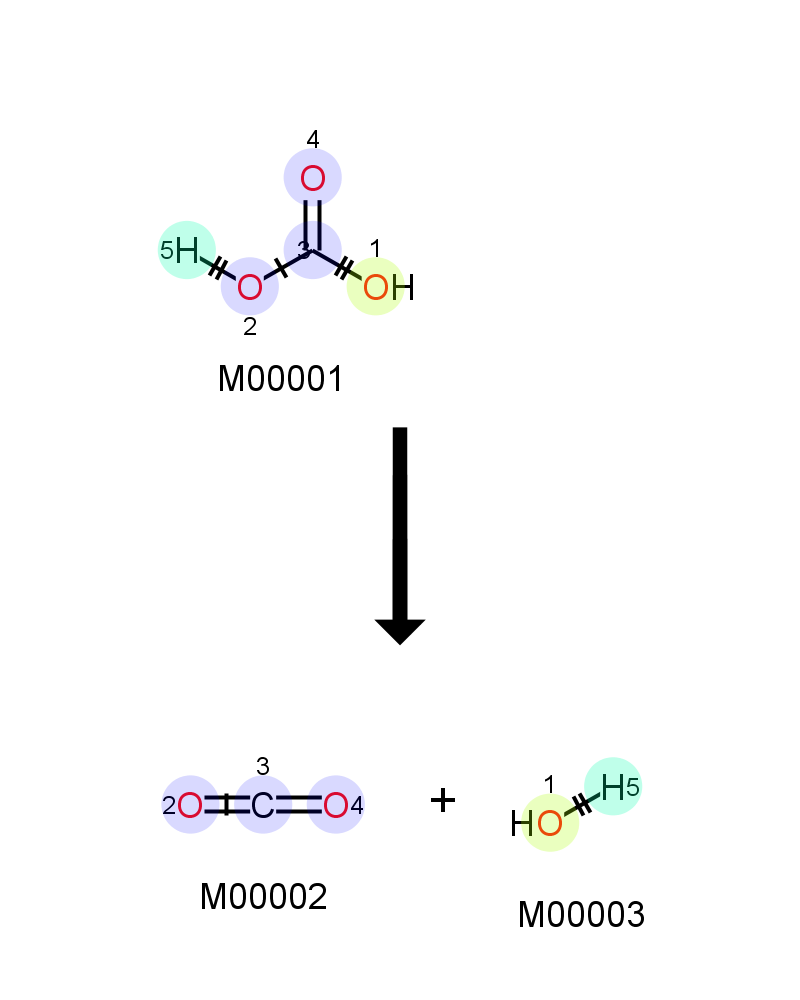

Supplement: Supplementary file 8 — Additional file 8. Zip-file containing all necessary data/files to run the software. [file 13321_2018_269_MOESM8_ESM.zip › demo/ReactionTemplates/R00010_R00132/ECBLAST_R00010_R00132_0_AAM.png]

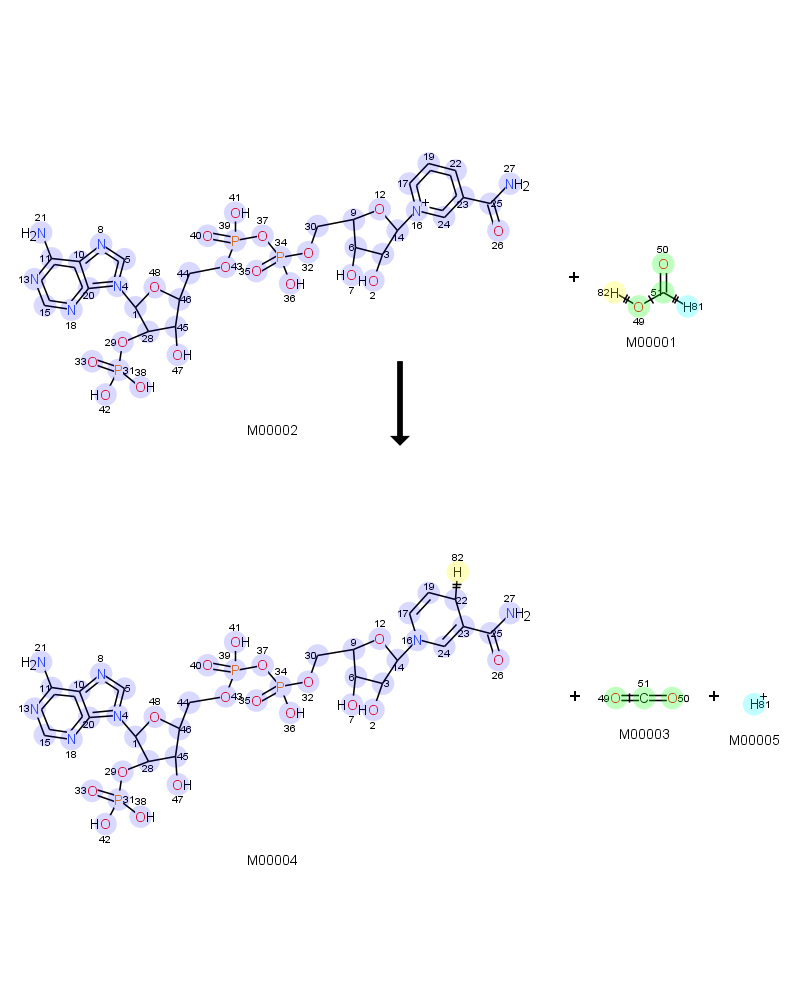

Supplement: Supplementary file 8 — Additional file 8. Zip-file containing all necessary data/files to run the software. [file 13321_2018_269_MOESM8_ESM.zip › demo/ReactionTemplates/R00011_R00134/ECBLAST_R00011_R00134_0_AAM.png]

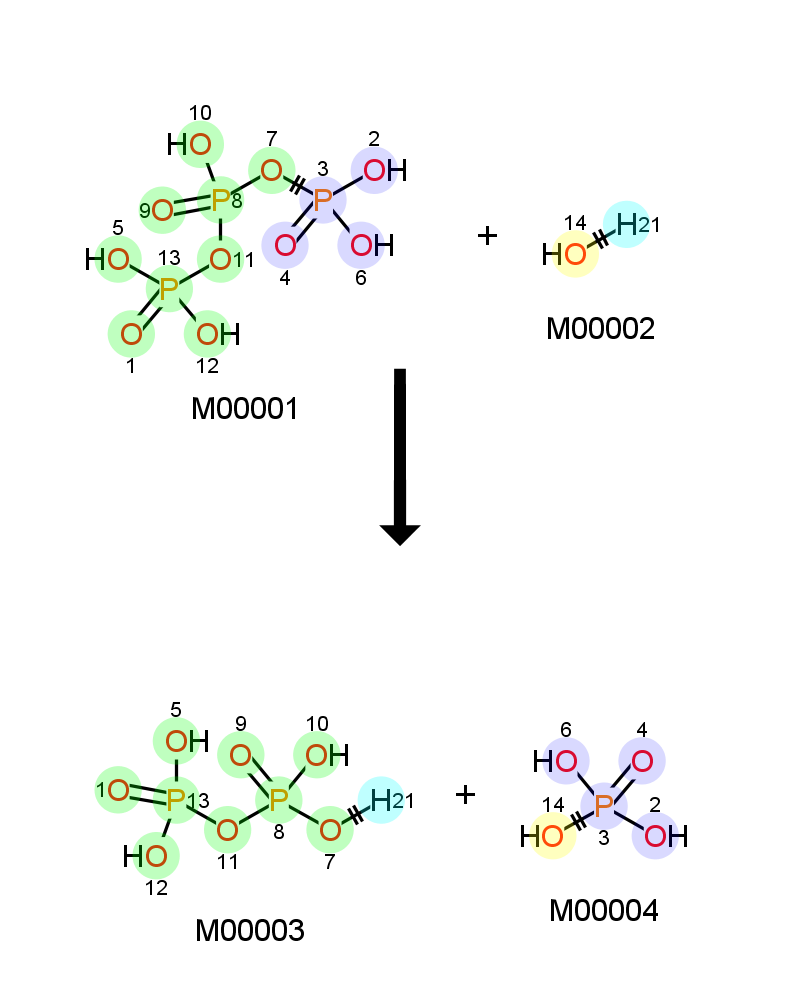

Supplement: Supplementary file 8 — Additional file 8. Zip-file containing all necessary data/files to run the software. [file 13321_2018_269_MOESM8_ESM.zip › demo/ReactionTemplates/R00012_R00138/ECBLAST_R00012_R00138_0_AAM.png]

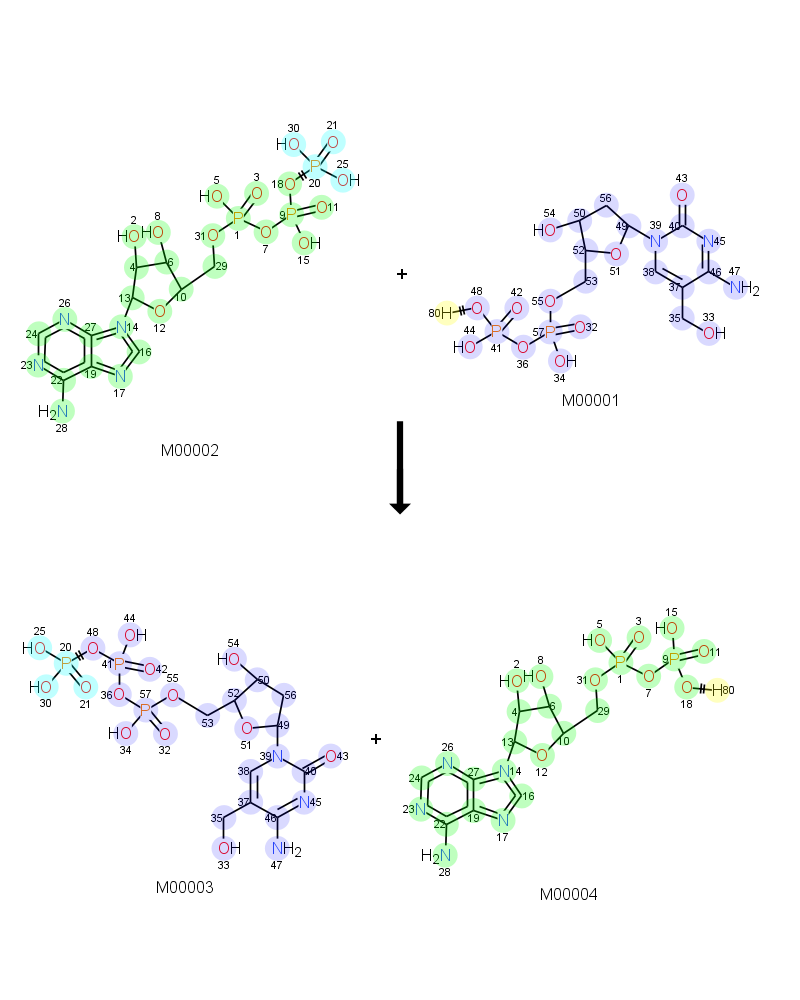

Supplement: Supplementary file 8 — Additional file 8. Zip-file containing all necessary data/files to run the software. [file 13321_2018_269_MOESM8_ESM.zip › demo/ReactionTemplates/R00013_R00139/ECBLAST_R00013_R00139_0_AAM.png]

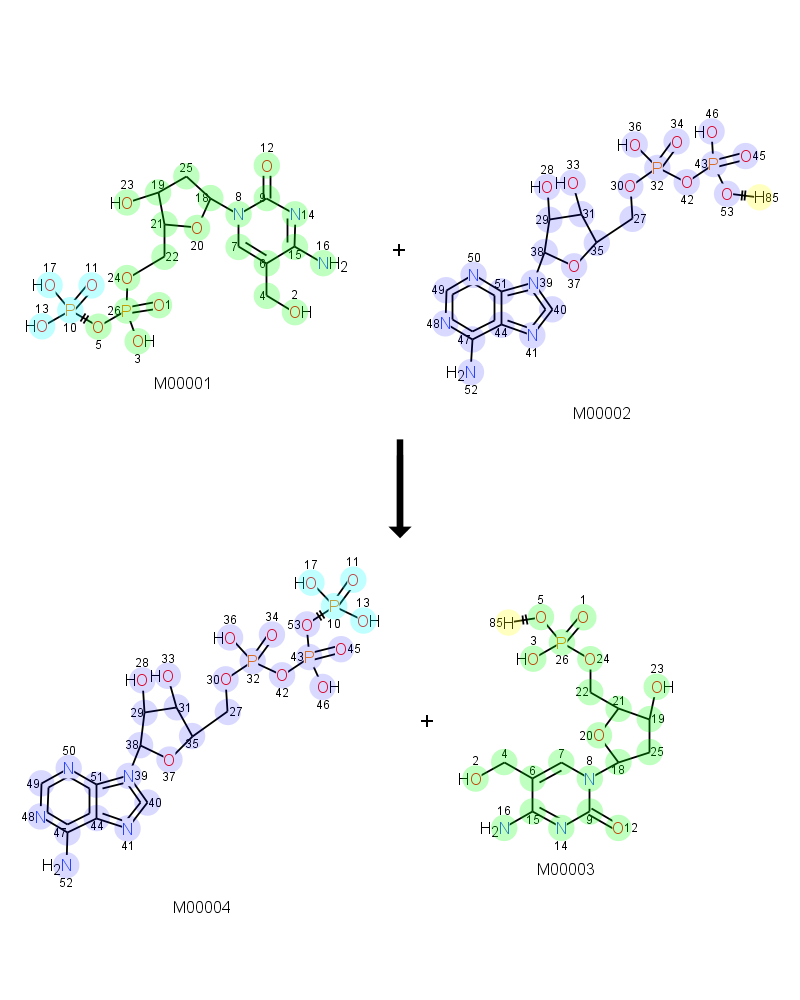

Supplement: Supplementary file 8 — Additional file 8. Zip-file containing all necessary data/files to run the software. [file 13321_2018_269_MOESM8_ESM.zip › demo/ReactionTemplates/R00014_R00140/ECBLAST_R00014_R00140_0_AAM.png]

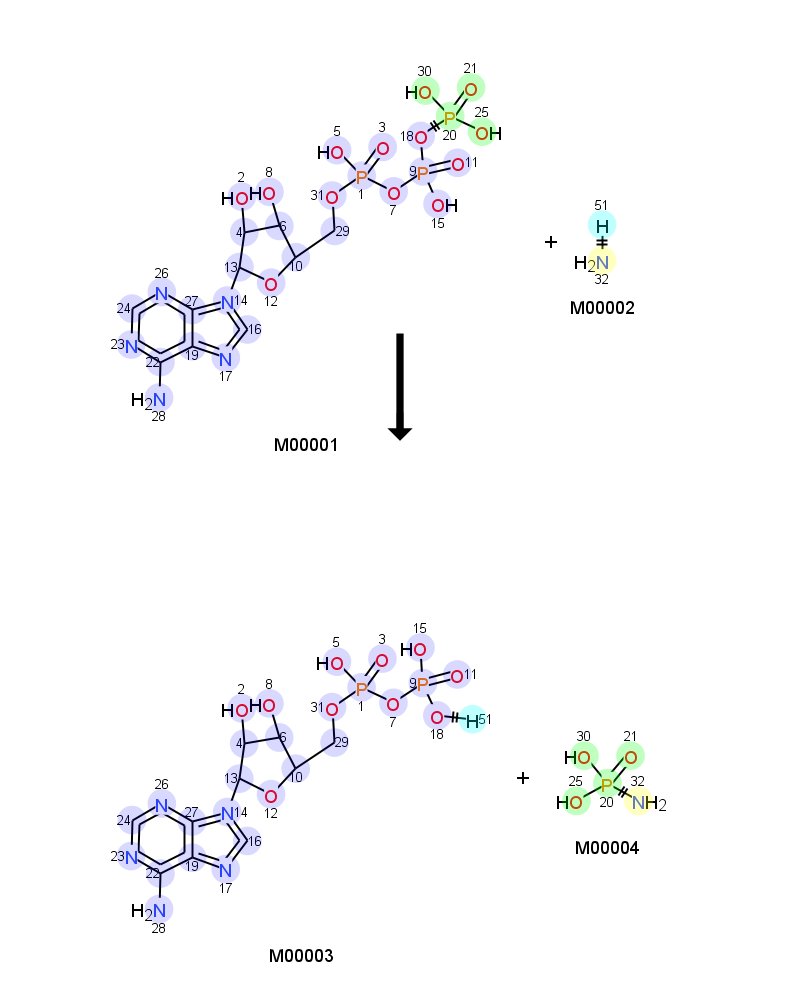

Supplement: Supplementary file 8 — Additional file 8. Zip-file containing all necessary data/files to run the software. [file 13321_2018_269_MOESM8_ESM.zip › demo/ReactionTemplates/R00015_R00141/ECBLAST_R00015_R00141_0_AAM.png]
