# Supplementary material for: Automated reaction database and reaction network analysis: extraction of reaction templates using cheminformatics
Source: J Cheminform. 2018 Mar 9;10:11. doi: 10.1186/s13321-018-0269-8 (PMC5845084; doi:10.1186/s13321-018-0269-8)
Supplement: Supplementary file 9 — Additional file 9. Zip-file containing the source code of the software. [file 13321_2018_269_MOESM9_ESM.zip › source_code/CDK/cdk/bin/org/openscience/cdk/iupac/parser/package.html]

The core classes of the ChemNomParse project, an open source attempt
of a chemical nomenclature parser, call via generate (name). See ChemNomParse Project Page for more information.
