# Supplementary material for: Automated reaction database and reaction network analysis: extraction of reaction templates using cheminformatics
Source: J Cheminform. 2018 Mar 9;10:11. doi: 10.1186/s13321-018-0269-8 (PMC5845084; doi:10.1186/s13321-018-0269-8)
Supplement: Supplementary file 9 — Additional file 9. Zip-file containing the source code of the software. [file 13321_2018_269_MOESM9_ESM.zip › source_code/CDK/cdk/nbproject/README.html]

NetBeans build system README


# README for NetBeans build environment.

## Philosophy This section discusses the rational behind the way the project files for NetBeans have been set up. The objectives were: - To allow users familiar with NetBeans to contribute to the CDK project without being obliged to learn Eclipse. - To leverage, as far as possible, the current build system. - To require no changes to the current build system (other than a few bug fixes). - To avoid, as far as possible, the necessity to maintain the NetBeans environment, since this is unlikely to be done by the main maintainers. - To cut build time to a minimum, without compromising the previous objective. - To support the "occasional" developer rather than one spending considerable time on the project, in particular favouring simplicity over competeness by omitting, for example, full support for those elements that require unusual building such as the smiles parser (uses javacc). These objectives have been addressed by adopting a two phase approach: 1. Perform a complete build of the CDK via the existing build file to act as a baseline. 2. Compile any changed files directly into a 'delta' directory and use these classes in preference to the ones in the baseline, with all other classes being taken directly for the baseline build. Quick Start In order to participate in the CDK developement process using NetBeans perform the following steps: 1. Run a recent version of NetBeans 2. Clone the CDK repository by clicking menu Team/Git/clone... 3. Select Git repository (at time of writing git://cdk.git.sourceforge.net/gitroot/cdk/cdk) 4. Select the branch(es) you wish to clone 5. Specify the destination directory. This is in two parts, the parent directory which could be where you normally keep your netbeans projects or somewhere new, and the project directory itself (CDK would be a good choice, but you can suit yourself - the project name will be CDK anyway). 6. Cup of coffee while it downloads (or lunch more like). 7. Right click in the Projects tab, open Project... then select the project directory. 8. Perform a baseline build by right clicking the project name (CDK) in the Projects tab and selecting CDK-rebuild. 9. Another cup of coffee or light snack (about 25 mins). 10. Make sure the build has worked. There will likely be lots of deprecated warnings etc but there should be no hard errors and the build output window should finish with the magic words "BUILD SUCCESSFUL". 11. You are now ready to roll. You can modify most source files and test files. You can get them built by selecting "build project" (F11) or you can just let them get built when you run a test. In order to run a test, right click on the test source and select "test file" or "debug test file". If there is a test file with the same name as a source file but with "Test" appended then you can also run it by right clicking on the source file and selecting the test option. 12. When you have all your changes debugged and finished, use Git to commit them locally. This can be done by clicking the project name (to act on the whole project) then selecting menu Team/Commit... and following the dialog. Deselect any files that you don't want to make part of this new version (if any) and check that all modified files are present, write a suitably descriptive message then go ahead. Be sure that you are ready - don't do a commit unless you are sure that everything is OK. 13. Generate a patch file. Menu Team/Export/Export Commit... then follow the dialog. Save the patch file (usually add extension .patch) somewhere you can get at. 14. Mail the patch to an appropriate maintainer to get them to check it out and then apply it to the master sources. 15. Repeat as desired... Implementation Details In order to determine whether a file has been changed since the last full build a timestamp is recorded in "nbproject/cdk-dynamic.properties". Files modified after this time are recompiled into the delta folder (netbeans-delta-build). Both main sources and test files are compiled into the same place for simplicity. The build target is referenced in all the run and test targets, so running a test for example will compile any needed sources first. Because of the reference to the baseline build this is usually very rapid (a few seconds). Normally it will not be necessary to invoke any of the targets in the ant files explicitly, with the possible exception of forcing the initial baseline build of the CDK, which it might be useful to perform at the users convenience (it takes around half an hour). This can be achieved by right clicking the project name in the Projects tab after first opening the project, and then selecting CDK-rebuild. The following elements have been added to the cdk: | | | | --- | --- | | nbproject/project.xml | This defines the project to NetBeans. Most of the actions associated with keys and menu items (test file, build etc) have been mapped to targets in the ant build file ide-file-targets. The java version used by the *IDE* to parse the java files is set here (though this isn't used to actually compile them). This file includes "build.props" from the main CDK to define the version information of this build. It also includes nbproject/CDK-build.properties to define the classpaths for checking and code completion. | | nbproject/ide-file-targets.xml | This is an ANT build file that defines all the targets to permit the IDE to perform builds, tests, debugs etc. It occasionally calls targets in the CDK build file, eg to rebuild the CDK from scratch. When it rebuilds the CDK it writes a tilmestamp to cdk-dynamic.properties, and if it finds the list of classpaths in CDK-build.properties out of date it will update it. The java version actually used for compilation is set here, so if it needs updating this must be done both here and in project.xml (there doesn't seem and easy way round this as project.xml doesn't allow the use of a property to define the version). See the commments in the file for info about the various targets. | | nbproject/CDK-dynamic.properties | This contains the timestamp of the last local build of the full CDK. It is ignored by GIT and recreated as needed by ide-file-targets. | | nbproject/CDK-build.properties | This contains defines that are common between ide-file-targets and project.xml, notably the compile/run classpaths. It is sometimes updated by ide-file-targets. | | netbeans-delta-build | This contains the compiled classes of the source files that have been modified since the baseline CDK build was made. It is created (and deleted) by ide-file-targets and is ignored by GIT. |
